# Significance of the Identification in the Horn of Africa of an Exceptionally Deep Branching *Mycobacterium tuberculosis* Clade

**DOI:** 10.1371/journal.pone.0052841

**Published:** 2012-12-27

**Authors:** Yann Blouin, Yolande Hauck, Charles Soler, Michel Fabre, Rithy Vong, Céline Dehan, Géraldine Cazajous, Pierre-Laurent Massoure, Philippe Kraemer, Akinbowale Jenkins, Eric Garnotel, Christine Pourcel, Gilles Vergnaud

**Affiliations:** 1 Univ Paris-Sud, Institut de Génétique et Microbiologie, UMR 8621, Orsay, France; 2 CNRS, Orsay, France; 3 Laboratoire de biologie clinique, hôpital d'instruction des armées Percy, Clamart, France; 4 Hôpital militaire Bouffard, Djibouti, Djibouti; 5 Hôpital d'instruction des armées Alphonse Laveran, Marseille, France; 6 Department of Veterinary Tropical Diseases, Faculty of Veterinary Science, University of Pretoria, Onderstepoort, Pretoria, South Africa; 7 DGA/MRIS- Mission pour la Recherche et l'Innovation Scientifique, Bagneux, France; St. Petersburg Pasteur Institute, Russian Federation

## Abstract

Molecular and phylogeographic studies have led to the definition within the *Mycobacterium tuberculosis* complex (MTBC) of a number of geotypes and ecotypes showing a preferential geographic location or host preference. The MTBC is thought to have emerged in Africa, most likely the Horn of Africa, and to have spread worldwide with human migrations. Under this assumption, there is a possibility that unknown deep branching lineages are present in this region. We genotyped by spoligotyping and multiple locus variable number of tandem repeats (VNTR) analysis (MLVA) 435 MTBC isolates recovered from patients. Four hundred and eleven isolates were collected in the Republic of Djibouti over a 12 year period, with the other 24 isolates originating from neighbouring countries. All major *M. tuberculosis* lineages were identified, with only two *M. africanum* and one *M. bovis* isolates. Upon comparison with typing data of worldwide origin we observed that several isolates showed clustering characteristics compatible with new deep branching. Whole genome sequencing (WGS) of seven isolates and comparison with available WGS data from 38 genomes distributed in the different lineages confirms the identification of ancestral nodes for several clades and most importantly of one new lineage, here referred to as lineage 7. Investigation of specific deletions confirms the novelty of this lineage, and analysis of its precise phylogenetic position indicates that the other three superlineages constituting the MTBC emerged independently but within a relatively short timeframe from the Horn of Africa. The availability of such strains compared to the predominant lineages and sharing very ancient ancestry will open new avenues for identifying some of the genetic factors responsible for the success of the modern lineages. Additional deep branching lineages may be readily and efficiently identified by large-scale MLVA screening of isolates from sub-Saharan African countries followed by WGS analysis of a few selected isolates.

## Introduction

Tuberculosis (TB) has occurred in humans for at least several thousand years. Archaeological findings from a number of Neolithic sites in Europe, pre-Columbian sites in South America and sites from ancient Egypt to the Greek and Roman empires showed typical skeletal changes associated with tuberculosis, at least some of which could be confirmed with ancient DNA analysis [1], [2], [3], [4], [5], [6], [7], [8], [9]. The progenitor species of the *M. tuberculosis* complex (MTBC) is unknown, as, in contrast with other pathogenic mycobacteria, no environmental source has been found so far for this human pathogen. One model is that all the members of the MTBC derived from an ancestor which might be related to “*Mycobacterium canettii*”, the smooth group of *M. tuberculosis* (SmTB). The first “*M. canettii*” strain was described forty years ago, and its relative position with respect to the MTBC is not yet fully established [10], [11], [12], [13], [14], [15], [16]. These isolates are exceptional in that their evolution is not clonal and they undergo recombination with unknown mycobacteria. Their morphotype (smooth colonies) is also very different and was the basis for their initial identification [17].

In contrast, the MTBC *sensu stricto* (i.e. not including “*M. canettii*”) is highly clonal. One hypothesis is that the apparent switch from non-clonality to clonality resulted from the emergence of the obligate pathogen with a restricted ecological niche including humans and some animal species [13], [17], [18] and limited opportunities for detectable recombination events [19]. During the past 20 years, and owing to the advent of molecular tools, some of which can be applied to routinely type thousands of isolates, our understanding of the population structure of the MTBC has increased tremendously. Different complementary methods have contributed to this knowledge: spoligotyping [20], IS6110 typing [21], Large-scale polymorphism (LSP) [10], variable number of tandem repeats (VNTR) typing [22], SNP analysis [23], partial or whole genome sequencing of selected representative strains [14], [24], [25]. Six main deep branching human-adapted lineages have been defined so far on the basis of canonical deletions and point mutations [15], [26], [27] or characteristic spoligotype signatures [18], [25], [28] and confirmed by partial or whole genome sequencing [14], [24], [25]. They can be further divided in strain families [14], [15], [18], [25], [26], [27], [28]. Lineage 1 contains the East-African-Indian (EAI) and some Manu spoligotype families, lineage 2 the Beijing group, lineage 3 the Central Asian (CAS) spoligotype family and lineage 4 the H/T, X and LAM families. Lineages 5 and 6 both corresponding to *Mycobacterium africanum* are observed predominantly and at very high frequency in Western Africa [29], [30], [31], [32]. Finally, there exist a number of animal associated lineages or ecotypes including *M. bovis*, *M. caprae, M. pinnipedii-M. microti*, *M. orygis*, *M. bovis* from antelopes (Dassie bacillus) and *M. mungi* [18], [33] which all belong to the same clade.

The clonal population structure of the MTBC complex implies that it emerged from a unique location. This location is currently unknown. The most recent common ancestor (MRCA) of the extant MTBC lineages is estimated to have lived some 5.000–40.000 years ago [19], [34], [35], [36]. The presence of most lineages in Africa makes this continent a likely place of emergence of the MTBC. More significantly perhaps, the vast majority of the “*M. canettii*” isolates collected so far (less than 70) originated from the Horn of Africa and the geographic origin of the others is uncertain [13]. Sub-Saharan Africa is also the region where the documented most recent common ancestor of the human population originated [37], [38], [39], [40]. Under this hypothesis, characterising the genetic diversity of MTBC strains isolated in Sub-Saharan Africa including the Horn of Africa might be of major importance in trying to reconstruct the emergence of the MTBC. In this way, one might expect to identify the ecotype as defined by Cohan and col. [41], [42] corresponding to the place of birth of the MTBC. This ecotype should be recognisable by its deep branching in the MTBC and by its restricted geographic spread. A study by Godreuil et al. investigated 62 isolates from patients from the Republic of Djibouti (Horn of Africa) by spoligotyping and multiple locus VNTR analysis (MLVA) using the MIRU-VNTR24 assay [22], [43]. All isolates could be assigned to either lineage 1, lineage 3 or lineage 4. This indicated that unknown deep branching *M. tuberculosis* geotypes, if they are present in Djibouti in spite of periodic selection [41], are rare. Hence a significantly larger number of isolates would need to be screened in order to identify them.

In the present investigation 435 *M. tuberculosis* isolates from the Horn of Africa (mostly collected in the Republic of Djibouti) have been genotyped, allowing the assessment of the current genetic diversity of *M. tuberculosis* in Djibouti and the identification of rare isolates of interest. Whole genome draft sequencing was applied to seven selected isolates, leading to the identification of a previously unknown and very deep lineage which is an excellent candidate for representing the original ecotype of the MTBC. The phylogenetic position of this lineage provides new insights on the early history of the MTBC.

## Results

### Genotyping and clustering of MTBC isolates

A total of 411 MTBC isolates, including two *M. africanum* and one *M. bovis*, were recovered over 12 years in Djibouti, the Republic of Djibouti. Together with 24 isolates from three neighbouring countries (Somalia, Sudan and Kenya) they were genotyped by spoligotyping and MLVA using the MLVA24_Orsay_ assay [13] and their susceptibility to four antibiotics was determined. Spoligotyping and MLVA defined respectively 80 and 211 genotypes, and a combination of the two methods led to 235 genotypes (eleven MLVA genotypes could be separated into 2 or 3 groups according to the spoligotype whereas 16 spoligotypes could be separated by MLVA). Lineage assignment was deduced on the basis of MLVA clustering and presence of spoligotype signatures [28], [44]. Of the 80 observed spoligotypes, thirty-three were classified as orphan by SITVITWEB and several were found mostly in East Africa or Saudi Arabia (Table S1).

**Figure 1:**
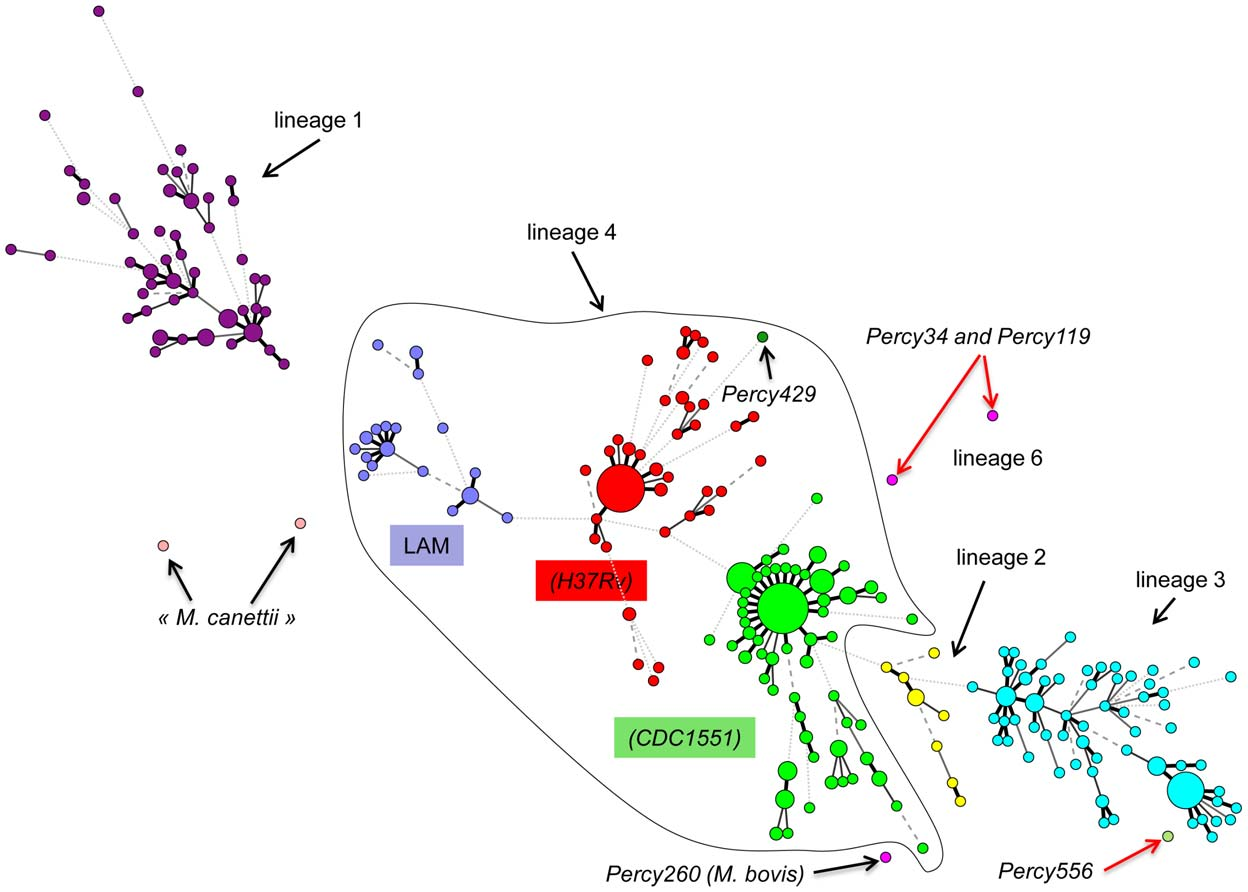
Minimum spanning tree representation of the clustering of 435 isolates from the Horn of Africa. The color code reflects the main MLVA clusters. Lineages (1 to 6) and some sublineages (CDC1551, H37Rv, …) are indicated. The size of the circles reflects the number of isolates with an identical genotype. Branches longer than 10 are not drawn. The main outlier candidates are arrowed (red arrows: isolates selected for sequencing).

The results of MLVA clustering for the 435 isolates are shown on Figure 1 in a condensed format using a minimum spanning tree (MST) representation and in Figure S1 as a dendrogram presenting the isolates in each cluster, their spoligotype, the Spoligo-international-type (SIT) when available from SITVITWEB and the clade (the same color code is used in both figures). Two “*M. canettii*” and two reference strains (H37Rv and CDC1551) were also included. The predominant human lineages are represented. Lineage 4 which includes two T/H subfamilies (colored in red and green) and the LAM subfamily (colored in dark blue) is the most abundant (252 isolates, 57.9%). Lineage 3 (CAS clade, colored in bright blue) is represented by 98 isolates (22.5%), lineage 1 (EAI clade colored in purple) by 70 isolates (16%) and lineage 2 (colored in yellow and including the Beijing clade) by 11 isolates (2.75%). The diversity inside lineage 1 (46 combined genotypes for 70 strains) is slightly higher than that of the lineage 4 (120 combined genotypes for 252 strains) and lineage 3 (CAS) (55 combined genotypes for 98 strains).

**Figure 2:**
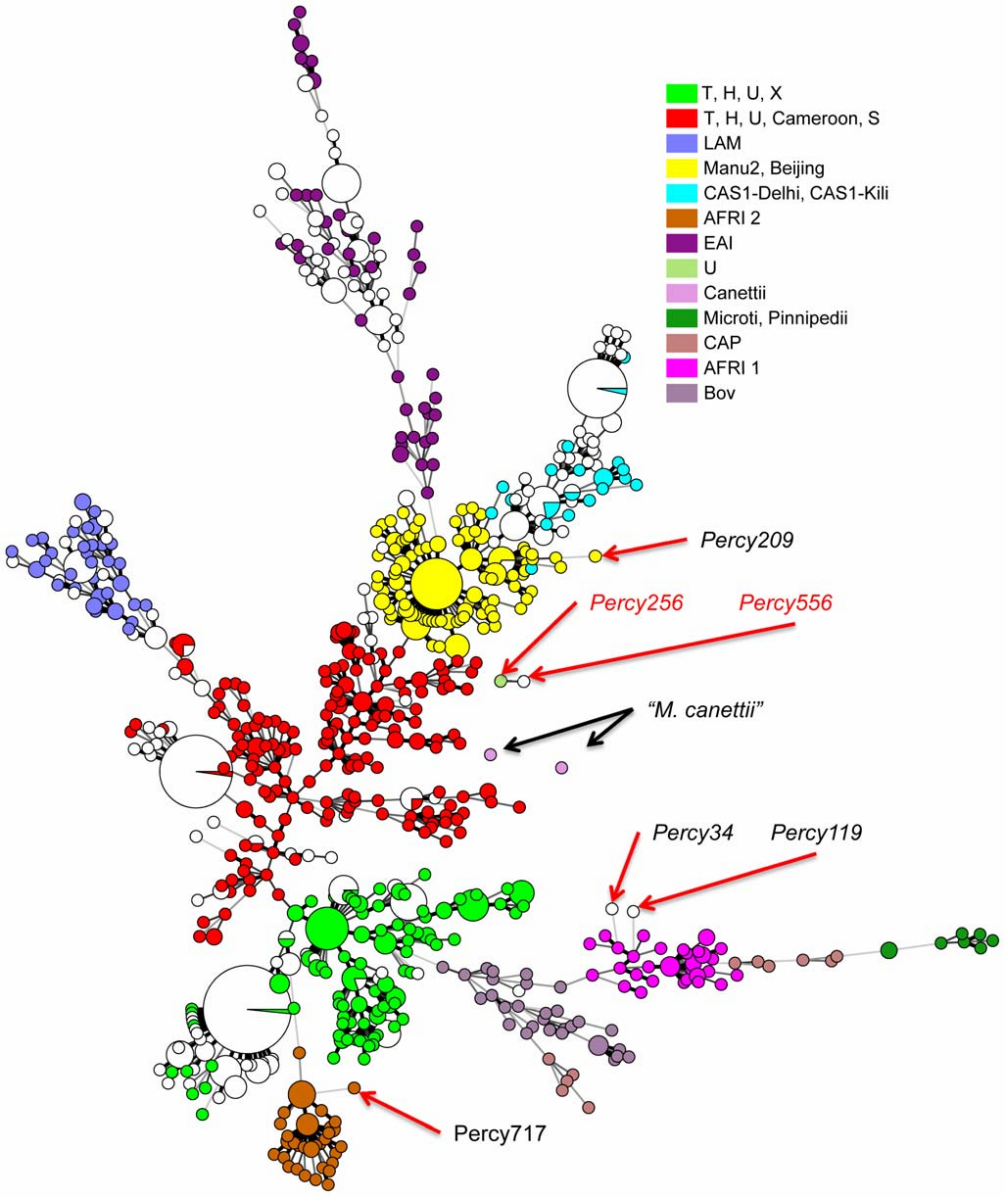
Minimum spanning tree representation of the Horn of Africa isolates with respect to 700 isolates of various origins. The 435 isolates from the Horn of Africa are displayed in white, whereas the isolates of worldwide origins are colored according to lineage using the same color code as in Figure 1. Red arrows: isolates selected for sequencing. The minimum spanning tree analysis is based upon the 19 VNTR loci shared by the 24 loci MLVA assays used by [13] and [22].

To get an idea of the diversity of our collection as compared to isolates from diverse origins worldwide, we performed an MLVA clustering using available data for 700 isolates from Europe, West Africa and Asia, including data from the 186 isolates in the VNTRplus database [45]. The MST in Figure 2 shows the MLVA clustering achieved when using the 19 loci shared by two 24-loci assays, MLVA24_Orsay_ [13] and MIRU-VNTR24 [22] (MLVA panels are recalled in Table S2). In this figure, the isolates from the present investigation are shown in white whereas the additional isolates are colored as indicated according to lineage and/or spoligotype clade. Most isolates from Djibouti do not differ significantly from the bulk but some large subclusters are more specifically found in Djibouti. Several isolates are distantly linked and they will be discussed in the following paragraphs.

### The lineage 4

Two hundred and fifty-two isolates belong to lineage 4, characterized by the absence of spacers S33–S36 in the DR locus. The larger cluster with 150 isolates includes reference strain CDC1551 (called here lineage 4-CDC and shown in bright green in Figures 1, 2 and S1) and the second with 78 isolates includes reference strain H37Rv (called here lineage 4-H37 and shown in red in Figures 1, 2 and S1). In these clusters there is no exact correlation between MLVA type and spoligotype except for some particular subclones. The most represented spoligotype is T1 (SIT53, 55 isolates), the very widespread lineage 4 ancestral form with the signature deletion of spacers S33–S36 (octal code 777777777760771), present in both 4-H37 and 4-CDC clusters.

In lineage 4-H37 (red), 38 isolates with the same MLVA genotype are separated into two groups by spoligotyping. Four isolates are SIT37 missing S13 (octal code 777737777760771), and thirty four have a spoligotype not present in SITVITWEB with an absence of contiguous spacers S4–S23 (octal code 700000017760771) which might be a one-step variant from SIT37 or SIT53. Interestingly this group contains only multi drug resistant (MDR) isolates collected across the whole sampling period (1999–2010, Figure S1) and may represent an ongoing outbreak.

In lineage 4-CDC, fifty-eight isolates with the same MLVA genotype show the ancestral SIT53 pattern or the T3-ETH (Ethiopia) SIT149 variant (additionally missing spacers S10 to S19, octal code 777000377760771). This predominant clone observed in 43 isolates was recovered along the whole sampling period and might also reflect the presence of an ongoing outbreak (Table S1).

Twelve among the 25 LAM cluster (dark blue) isolates show the spoligotype LAM09 having the characteristic LAM signature, absence of spacers S21 to S24 (SIT42, octal code 777777607760771). Seven others are LAM01 (SIT20 additionally missing spacer S3, octal code 677777607760771).

Isolate Percy429 (Figure 1, dark green) is weakly connected by MLVA to the lineage 4-H37 group. Its spoligotype is unique (del S4–S24, octal code 700000007760771) but shows the lineage 4 spoligotype signature, i.e. absence of spacers S33–S36.

### The lineage 3 (CAS) and lineage 2 (including Beijing)

The 98 CAS strains isolated in Djibouti (colored bright blue in Figure 1 and Figure S1) are separated into two groups by MLVA, one of which showing little diversity with 35 SIT21 (CAS-Kili) isolates and one variant. The second group contains 14 SIT26 (CAS1-Delhi, octal code 703777740003771) founder genotype isolates, and variants of SIT26: SIT25 missing the extra spacers S37 and S38 (16 isolates), SIT247 (5 isolates) and 6 other spoligotypes (12 isolates). Ten isolates from Sudan cluster in the CAS-Delhi group. Again in this clade the founder genotype is one of the most abundant genotypes.

The eleven lineage 2 isolates belong to the Beijing-family with a classical spoligotype (deletion S1 to S34, octal code 000000000003771) whereas 7 MLVA genotypes are observed. When clustered with 126 Beijing isolates from China, Thailand and Cambodia they localise into two groups together with isolates showing identical genotypes (data not shown). We searched for Beijing clade specific deletions and found that all 11 isolates were deleted for RD150, RD181 and RD142 demonstrating that they belong to the same currently highly successful Beijing sublineage. All the Beijing isolates were MDR.

### Lineage 1, lineage 6 and Percy556

The cluster colored in purple in Figure 1 and Figure S1 shows the 70 EAI isolates. Three main subgroups are observed, the larger one including essentially isolates from Djibouti and the Horn of Africa (as shown by comparison with other isolates worldwide, Figure 2) with a spoligotype identical or similar to EAI8-MDG (SIT109 Madagascar). A second group containing mostly isolates from Djibouti shows a spoligotype close to EAI-SOM (SIT48 Somalia). The variability inside the EAI clade is large with 18 spoligotypes and 43 MLVA types for a total of 70 isolates. The other lineage 1 clade, represented by the Manu1, 2, 3 spoligotype is not detected [46].

The two *M. africanum* isolates from Djibouti (Percy34 and Percy119) cluster with our collection of 81 *M. africanum* isolates from West-Africa in an outgroup position of lineage 6 West African 2 (Figure 2, two open circles within the pink cluster). Their spoligotype shows the canonical deletion S39 but interestingly S8 is present whereas *M. africanum* are frequently deleted of S7 to S9 [18], [47]. The unique *M. bovis* isolate does not show any particularities when compared to 30 other *M. bovis* isolates.

Lastly, one isolate, Percy556 isolated in 2007 could not be strongly linked to any lineage (Figure 1 and Figure 2). Percy256, another isolate from the Percy collection (recovered in France in 1998 from a patient of unknown origin [48]) clusters closely with Percy556 by MLVA (Figure 2, arrowed). Similar to EAI lineage 1 isolates, and in contrast with lineages 2 to 6, their genome is intact for regions of deletion TbD1 and RD9. In both isolates the number of repeats at VNTR Miru26 is 5 whereas it is 2 in all tested EAI isolates. Portions of the *katG* and *gyrA* genes were sequenced showing that they belong to Principal Genetic Group (PGG) 1 [35]. Finally both isolates show an identical spoligotype (SIT910, octal code 700000007177771). In particular they possess spacers S34 and S39 which is a feature found in some lineage 1 isolates [49] and some rare Beijing-family ancestor isolates [50] in relation with the Manu ancestor lineage [46]. SIT910 spoligotype is very rare as it is represented by only three isolates in the SITVITWEB database, two from Ethiopia and one from the USA.

### Whole genome SNP analysis

**Figure 3:**
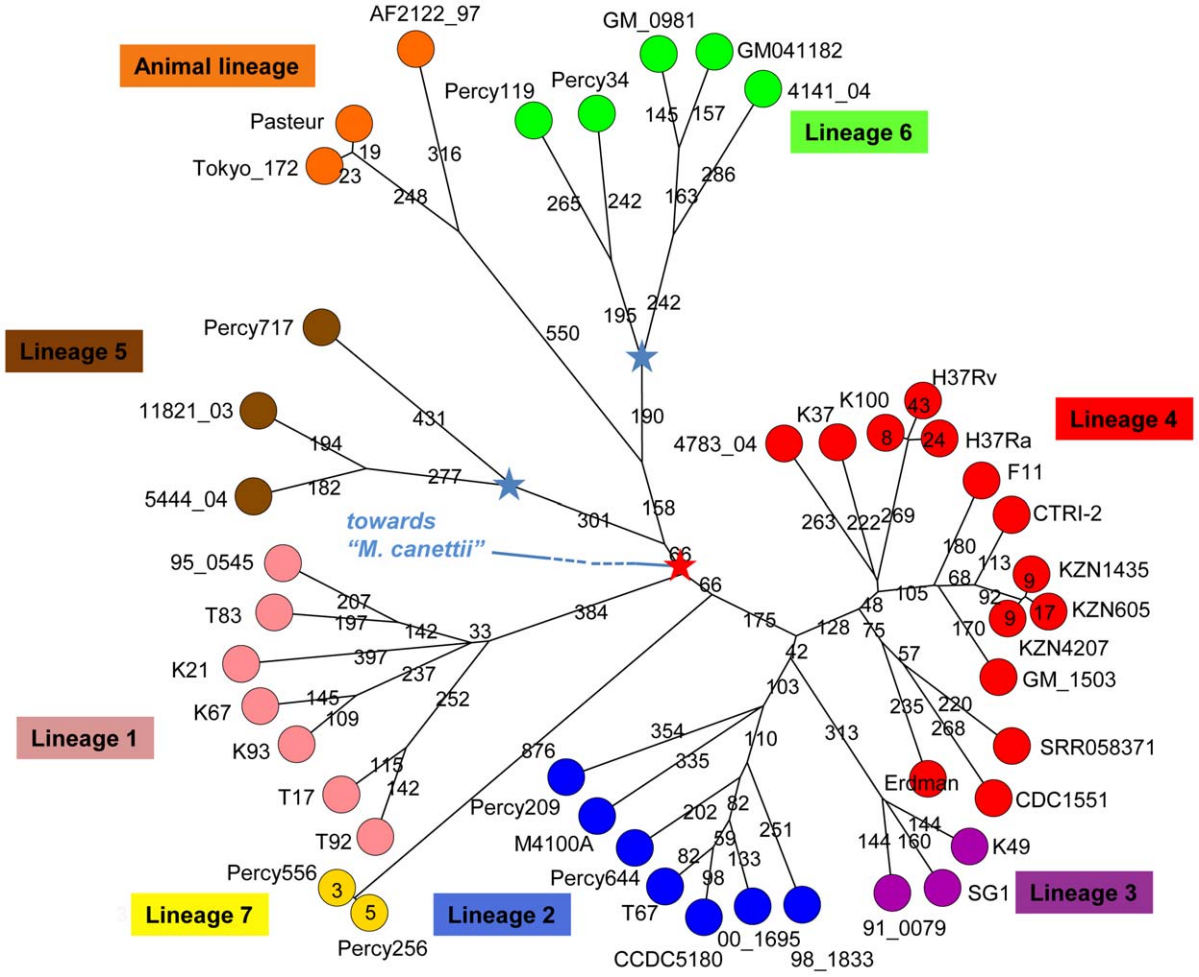
Minimum spanning tree based upon whole genome SNP analysis. The tree is based upon 13382 SNPs. The tree size is 13463, i.e. it contains approximately 0.6% of homoplasia. The length of each branch expressed in SNP numbers is indicated. The red star marks the approximate branching point of the *M. canettii* lineage according to [19]. The two blue stars indicate the positions of newly defined ancestral nodes within the two Africanum lineages 5 and 6.

Most isolates from Djibouti fit into well characterised lineages in agreement with previous reports [43]. However a few isolates appear to be only weakly connected to previously known lineages as shown in Figure S1 and Figure 2. This is the case for *M. africanum* Percy119 and Percy34 (weakly linked to lineage 6, Figure 2), and especially for Percy556. We performed draft whole genome sequencing in order to more precisely locate these isolates within the MTBC complex. Isolates Percy717, an *M. africanum* isolate from Gabon weakly connected to lineage 5 West African 1, Percy209 an isolate from a patient originating from Mali and Percy256 closely related to Percy556 were also included (Figure 2). Percy209 shows a SIT523 spoligotype (Manu ancestor spoligotype with all 43 spacers present) and is connected to lineage 2 (Figure 2). Percy644 a Beijing clade isolate was incorporated as a control. The seven isolates were positioned with respect to 38 strains including 14 fully sequenced genomes (Table 1). By mapping each dataset against the H37Rv genome, 13382 SNPs were identified, corresponding to 13358 positions in the H37Rv genome (at 24 positions, three states are observed, as can be deduced from Table S3 so that the number of distinct SNPs is 13382). Figure 3 shows a MST deduced from Table S4 SNP data and allowing the creation of hypothetical missing links. The size of the minimum spanning tree is 13463, an excess of 81 corresponding to an homoplasia level of 0.6%. Fifty-six SNPs are responsible for the homoplasia. Forty-three occur only twice, but 13 SNPs are responsible for almost half of the homoplasia. SNP s1319 in Rv0280 (a PPE family protein) occurs in seven positions in the MST. Rv2082, annotated as hypothetical protein in H37Rv refseq NC_000962.2 contains 5 homoplasic SNPs accounting for 14 out of the 81 excess. The red star in the central position of the Figure 3 MST indicates the approximate position of the MRCA for the MTBC complex, according to Namouchi et al. [19], i.e. the branching point of the “*M. canettii*” taxon, under the hypothesis that “*M. canettii*” can indeed be considered as an outgroup. Three branches were observed to radiate from this red star; one corresponds to lineage 1 including EAI (pink), a second one leads to lineages 5, 6 and the animal lineage including *M. bovis* (brown, green, orange), and the third one leads to lineages 2, 3 and 4 (blue, purple, red). Lineages 2–3 and lineage 4 split at a distance of 241 SNPs from the red star. The secondary split between lineages 2 and 3 occurs very shortly afterwards, 42 SNPs later. The distance from the red star to the tip of the branches is closely related within each lineage and across lineages: it is approximately 700–800 SNPs long for lineages 1 to 5, a little more than 900 SNPs for lineage 6 and 7, and a little more than 1000 SNPs in the animal lineage (Table 1). The Percy644 control strain fits in the lineage 2 (including the Beijing clade) as expected. It contributes to 1.5% of the size of the MST (202 bp terminal branch). Similarly, Percy717, Percy34 and Percy119 belong to their predicted lineages, 5, 6 and 6 respectively and they contribute to 8.4% of the total MST. Interestingly these two groups of strains define two new deep branching nodes and significantly older MRCAs within these lineages (indicated by blue stars in Figure 3). These nodes are located approximately midway along the lengthy initial lineage 5 and lineage 6 branches. Percy209 contributes a 354 SNPs terminal branch corresponding to 2.6% of the MST. Percy209 from Mali and M4100A from South Korea both showing a Manu ancestor SIT523 spoligotype split simultaneously from the rest of lineage 2 leading to the Beijing sublineage.

**Table 1:** Total branch length from central node to the tips.

strain	lineage	origin	reference	Average mapped sequencing depth	Reads size	Number of reads	Unambiguous bases	Percent genome coverage	distance to H37Rv	distance to origin*
95_0545	1	Laos	[14]	84.57	50	7282727	4305774	97.6	1517	766
K21	1	Zimbabwe	[14]	107.55	50	9349705	4346684	98.53	1565	814
K67	1	Comoro Islands	[14]	104.4	50	8966783	4294343	97.34	1550	799
K93	1	Tanzania	[14]	102.51	50	8861777	4322259	97.98	1514	763
T17	1	The Philippines	[14]	83.96	50	7259571	4323254	98	1502	751
T83	1	Vietnam	[24]	76.67	50	6307452	4113632	93.25	1529	756
T92	1	The Philippines	[14]	50.45	50	4284945	4246376	96.26	1507	778
CCDC5180	2	China	NC_017522.1	74.81	75	4364593	4375816	99.19	1016	766
00_1695	2	Japan	[14]	93.49	50	8025905	4292463	97.3	1012	742
98_1833	2	China	[14]	80.84	50	6960877	4305526	97.6	998	747
M4100A	2	South Korea	[14]	44.15	50	3767815	4266934	96.72	993	724
T67	2	China	[14]	89.61	50	7701364	4297050	97.4	1035	750
Percy209	2	Djibouti	[76]	191.56	100	8347754	4357804	98.78	1019	743
Percy644	2	Djibouti	[76]	92.26	75	5308661	4315553	97.82	1011	729
91_0079	3	Ethiopia	[14]	80.83	50	6965605	4308731	97.67	1033	740
K49	3	Tanzania	[14]	109.78	50	9491499	4323074	97.99	1009	748
SG1	3	India	[24]	43.36	75	2262749	3914082	88.72	1017	764
CDC1551	4	USA	NC_002755.2	74.53	75	4361677	4388976	99.49	782	769
4783_04	4	Sierra-Leone	[14]	86.98	50	7467143	4292623	97.3	589	712
GM_1503	4	The Gambia	[14]	96.43	50	8268654	4287553	97.19	617	700
K100	4	East Asia	[24]	44.27	50	3803577	4295974	97.38	51	716
K37	4	Uganda	[14]	103.23	50	8823216	4273457	96.87	548	671
CTRI-2	4	Russia	NC_017524.1	74.81	75	4372889	4383898	99.37	628	711
Erdman	4	USA	AP012340.1	74.67	75	4353796	4373213	99.13	692	679
F11	4	South Africa	NC_009565.1	74.95	75	4392684	4395734	99.64	619	702
H37Ra	4	USA	NC_009525.1	75	75	4409081	4409277	99.95	69	730
H37Rv	4	USA	NC_000962.2	75	75	4411640	4411532	100	0	751
KZN1435	4	South Africa	NC_012943.1	74.77	75	4374267	4387801	99.46	625	708
KZN4207	4	South Africa	NC_016768.1	74.75	75	4373030	4387874	99.46	616	699
KZN605	4	South Africa	NC_018078.1	74.78	75	4375131	4387947	99.47	633	716
SRR058371	4	Canada	[52]	101.3	50	8899513	4392486	99.57	734	721
11821_03	5	Sierra-Leone	[14]	91.64	50	7890161	4304782	97.58	1589	838
5444_04	5	Ghana	[14]	95.36	50	8191687	4294937	97.36	1577	826
Percy717	5	Djibouti	[76]	236.03	75	13590158	4318394	97.89	1549	798
GM041182	6	The Gambia	[32]	99.09	75	5716368	4326621	98.08	1602	976
4141_04	6	Sierra-Leone	[14]	68.74	50	5869294	4268935	96.77	1727	942
GM_0981	6	The Gambia	[14]	82.85	50	7098372	4283740	97.1	1693	964
Percy34	6	Djibouti	[76]	51.23	75	2890448	4231179	95.91	1715	851
Percy119	6	Djibouti	[76]	119.41	75	6771989	4253363	96.41	1625	874
Percy256	7	Djibouti	[76]	210.16	100	9139161	4348608	98.57	1566	947
Percy556	7	Djibouti	[76]	268.37	75	15319715	4281284	97.05	1564	945
Pasteur_1173P2	bovis_BCG	France	NC_008769.1	74.4	75	4210871	4244811	96.22	1792	1041
Tokyo_172	bovis_BCG	Japan	NC_012207.1	75.23	75	4317828	4304764	97.58	1796	1045
AF2122_97	bovis_BCG	Great Britain	NC_002945.3	74.56	75	4292706	4317765	97.87	1841	1090

* the average distance to central node 7 is 775 SNPs (standard deviation 23) in lineage 1, 743 (SD 14) in lineage 2, 750 (SD 12) in lineage 3, 713 (SD 25) in lineage 4, 820 (SD20) in lineage 5, 921 (SD 55) in lineage 6, 946 (SD 1) in lineage 7, 1058 (SD 27) in *M. bovis* strains.

As initially suggested by MLVA and spoligotyping, Percy256 and Percy556 are very exceptional compared to all the other studied genomes. They introduce a 876 SNPs long terminal branch to the tree, which contributes to 6.5% of the MST. This remarkable branch splits from the branch leading to lineages 2, 3, and 4 only 66 SNPs away from the red star. Previous investigations identified seven interruptions of coding sequences (ICDS), numbered ICDS0013, 0037, 0038, 0045, 0055, 0073, 0078, which occurred along superlineage 2-3-4 before the split between lineage 4 and lineages 2–3 [51]. ICDS0045 could not be analysed in Percy256 and Percy556, whereas ICDS0013 (Rv0618-Rv0619) and ICDS0038 (Rv1549-Rv1550) are present in Percy256 and Percy556. The four others are still intact in both strains. We propose to call this new lineage, lineage 7. Percy256 and Percy556 isolated respectively in 1998 and 2007, are separated from each other by only 8 SNPs.

### Rooting the MTBC

**Figure 4:**
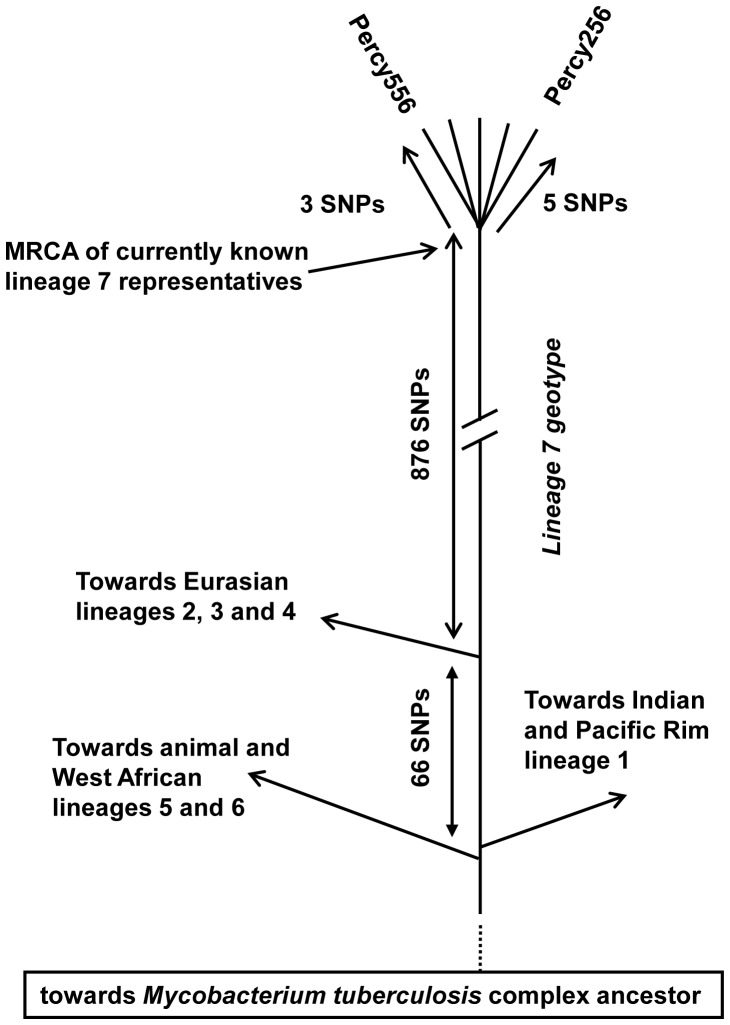
Schematic representation of the main historic events along the lineage 7 and Percy256-Percy556 associated geotype. The evolution of lineage 7 is displayed in a linear fashion from the *M. tuberculosis* ancestor (the obligate human pathogen in contrast to its environmental unknown *Mycobacteria* progenitor) to the Percy256-Percy556 geotype representative. The relative timing of the different splits is indicated. The hypothetical temporal succession of the split of the two Ancestral superlineages indicated here is suggested by the slightly abnormal mutation pattern along branch (6,7). More precise rooting of the MTBC will be needed to test this hypothesis.

Lineage 7 is unique by being the only lineage with a geographic location apparently limited to the Horn of Africa and as such coincident with the “*M. canettii*” lineage. It is also quite remarkable by its rarity. This strongly suggests that its current members constitute the geotypes resulting from the evolution of the *M. tuberculosis* ancestor in its original geographic location. Figure 4 draws a linear tree running from the putative ancestral human obligate pathogen to Percy256 and Percy556, as can be deduced from the MST in Figure 3. In this view, all contemporary worldwide lineages and sublineages are the result of three independent “Out of the Horn of Africa” events. The first two, corresponding to lineage 1 on one hand, and lineages 5, 6 and *M. bovis* on the other, are coincident or almost coincident. The third superlineage, subsequently leading to lineages 2, 3 and 4, emerged 66 SNPs later.

**Figure 5:**
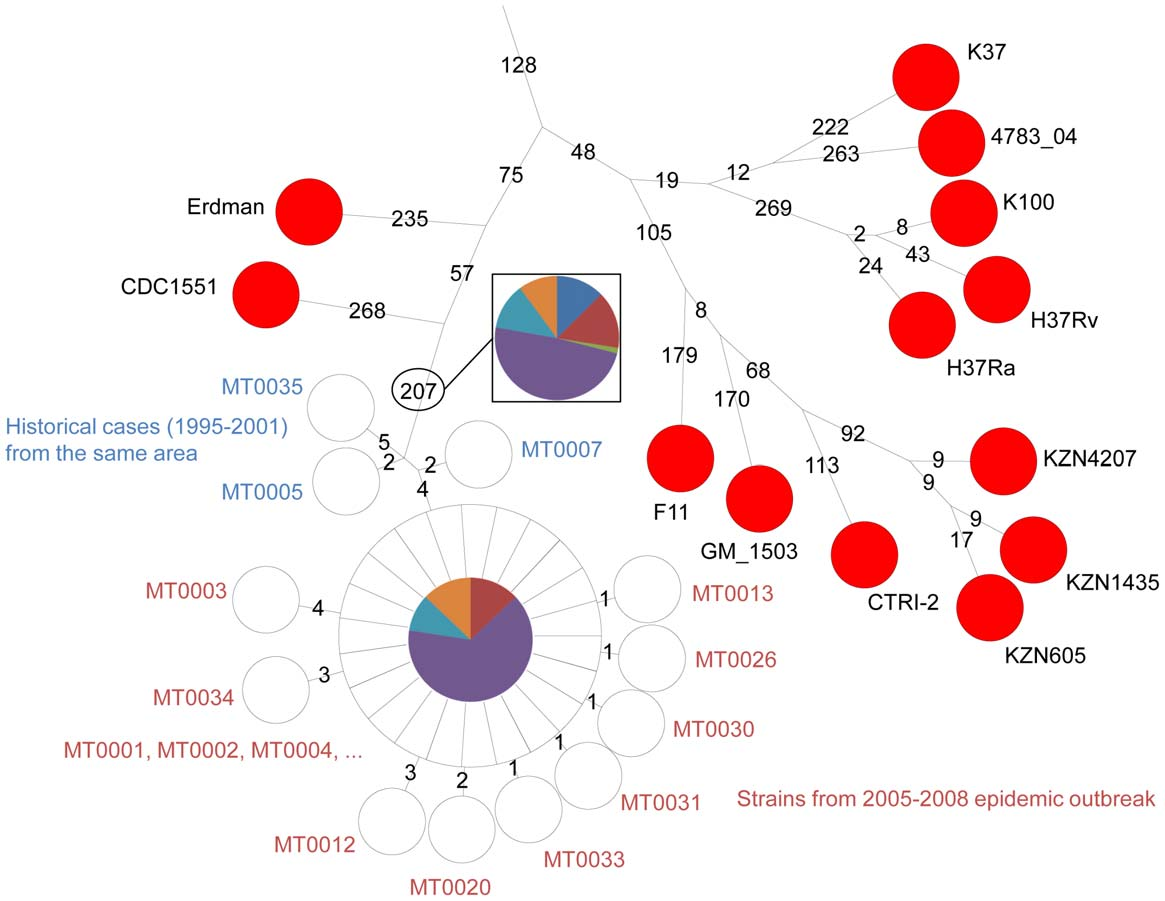
Genetic distances within an outbreak. Investigation of 32 *M. tuberculosis* outbreak isolates sampled in 2005–2008 and 3 historical isolates sampled in 1995–2001 from the investigation by [52]. The sequence reads were analysed together with the other genomes using the same SNP selection rules. The three historical samples collected a few years earlier in the same region are numbered in blue. Twenty-two outbreak samples corresponding to the central node show an identical genotype. Nine samples numbered in red are one to four SNPs away from the central node in a star like pattern. MT0001 the most likely index case belongs to the central node. Each branch length (number of SNPs) is indicated in black, logarithmic scaling is used. The proportion of the different mutations detected among historical and outbreak isolates is shown using the same color code as in Figure 7.

We attempted to estimate the time span which may correspond to these 66 SNPs. For this purpose, we took advantage of the previous investigation by Gardy et al. who sequenced 32 isolates from an outbreak, and additional historical isolates from the same region, collected a few years before [52]. We re-analysed the raw sequence reads applying the same SNPs detection procedure as above. Figure 5 shows the results obtained in the form of a MST. The outbreak isolates complex collected over three years contains 17 SNPs: 23 outbreak isolates show an identical genotype, whereas the other 9 are disposed in a star-like pattern around the central and presumably founder genotype, at a distance of 1 up to 4 SNPs. The historical isolates are defining a very close ancestral node. This provides a rough estimate of the MTBC branches growth rate, of approximately 1 SNP per 10 years in an outbreak context, which would convert into 10 thousand years for the whole MTBC, in reasonable agreement with independent estimates. It also indicates that lineage 1 and lineages 5–6-animal might have emerged within a single outbreak, whereas lineage 2-3-4 emerged a few centuries later.

### Regions of deletion along lineage 7 (Percy256 and Percy556)

To further explore the Percy556 genome, we aligned it against H37Rv and predicted the absence in Percy556 of 13 regions of more than 2 kb (Table S5). They were given arbitrary names according to their position in the list of missing regions larger than 1 kb. Two of them encompass phage-like elements: Del62_(1779276, 1788519) which is flanked by 12 bp direct repeats and Del103_(2973436, 2980972) containing phiRv2 prophage genes flanked by 28 bp repeats. Del97_(2969985, 2972110) encompass the tRNA^Val^ locus and an integrase. Eight missing regions correspond to genes encoding PPE or PE_PGRs family proteins. Deletion Del79_(2211370, 2219425) encompass 8 kb of the mammalian cell entry (Mce) operon 3 (*mce3*) including the *mce3B*, *mce3C*, *mce3D* and *mce3F* genes (Figure 5). Del79 is within the 12.7 kb RD7 region previously shown to be absent from *M. bovis* and lineage 6 West African 2 strains (spoligotype AFRI1) [10], [53]. In order to more precisely define the junction, we performed an assembly of reads mapping in the interval corresponding to the deletion and found a 1128 bp fragment which is present in genomes representing lineage 1, 2 and 3 and in the “*M. canettii*” CIPT140010059 (accession number refseq NC_015848.1) genome but absent in the lineage 4 genomes. The last deletion Del154_(3884925, 3887201) of approximately 2.2 kb encompass genes putatively involved in metabolic pathways, Rv3468 (involved in dTDP-L-Rhamnose biosynthesis), Rv3469 (aromatic hydrocarbon degradation) and Rv3470 (valine and isoleucine biosynthesis).

**Figure 6:**
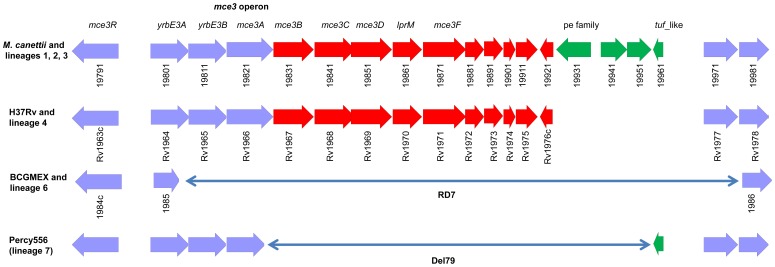
comparison of the Percy556 *mce3* deletion 79 with the organisation of the operon in H37Rv, in “*M. canettii”* strain CIPT140010059, and in *M. bovis* BCG. Four different organizations are observed. The most parsimonious explanation is that the *M. canettii* configuration represents the ancestral situation from which 3 different deletions are derived via at least 2 independent deletion events.

To check the distribution of Del79 and Del154, we selected oligonucleotide primers inside the region of deletion and in flanking regions and we performed a PCR amplification on genomic DNA samples belonging to the different lineages. Del79 was tested using primers inside Rv1971 (*mce3F*) and no amplification was observed in Percy556 and Percy256. The same results were obtained with Percy34 and Percy119, the lineage 6 *M. africanum* isolates which is not unexpected given the overlap of Del79 with the RD7 deletion (data not shown and Figure 6). Using primers flanking the deletion a 1.6 kb product was obtained in Percy556 and Percy256 but as expected not in any other isolate including two “*M. canettii*”. Sequencing of this fragment confirmed the extent of the deletion as compared to H37Rv and showed the presence of a *tuf*-like gene and a non-coding fragment both absent from the H37Rv genome and present in genomes of lineages 1, 2 and 3. Del154 was tested using primers inside Rv3468 and a product of 340 bp was observed in every sample except Percy556 and Percy256.

### Mutation patterns and location of the ancestral node

**Figure 7:**
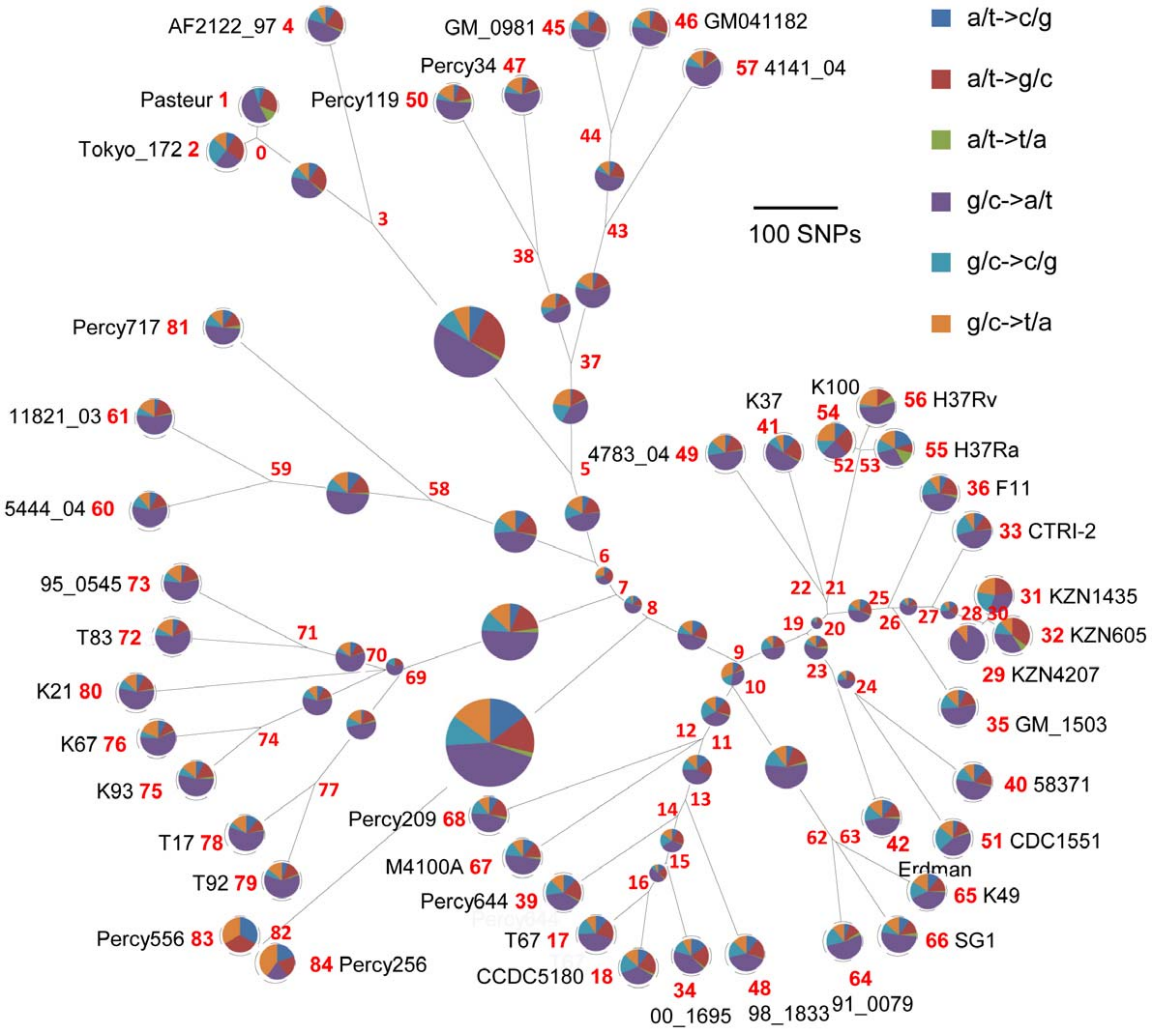
Mutation ratio along the MTBC complex. The proportion of each mutation type on each branch of the Figure 3 minimum spanning tree was calculated. The disk size reflects the branch length except for terminal branches (leaves) for which identical size disks are used. The mutation direction is deduced by comparing the genotypes of the hypothetical nodes (numbered in red) and considering node 7 as the ancestral node. The color code and branch scale are indicated. The disk on branch (6,7) is slightly abnormal, suggesting that the MTBC MRCA is located somewhere along this branch rather than coincident with node 7.

Ford et al. have described a strong GC to AT bias in new mutation events in *M. tuberculosis* [54] which they hypothesize may reflect the role of oxidative damage in the evolution of *M. tuberculosis*. We analysed and compared the mutation types in each branch of the Figure 3 minimum spanning tree. The results are presented in Figure 7, in which each node is numbered and the relative proportion of the different mutations is shown by disks under the assumption that node 7 is the ancestral node. The disk of the terminal branches (leaves) are shown with an equal size independently of the branch size, whereas internal disks sizes are proportional to branch length. The previously reported large excess of G/C→A/T mutations are illustrated here with no significant differences between internal and terminal branches across the whole tree. This indirectly confirms the ancestral status of node 7: selecting any other node (with the exception of node 6) as ancestor would create strikingly different mutation pattern along some branches compared to the others. Interestingly, the pattern of mutations along branch (6,7) is slightly abnormal compared to the others, with a deficit of G to A and an excess of A to G mutations which might indicate that the more precise position of the root of the MTBC is located somewhere along this branch. Lineage 7 shows a very similar pattern compared to other branches. Considered collectively, the 30 SNPs distinguishing the outbreak isolates and close neighbors in Figure 5 also show a similar pattern.

## Discussion

### MLVA allows the identification of rare and phylogenetically important isolates

Comas et al. recently argued that VNTR-based genotyping could not perfectly define phylogenies in MTBC as compared to single nucleotide polymorphism or large sequence polymorphism for phylogenetic studies [25]. Indeed the homoplasy level is higher with VNTRs as compared to carefully selected SNPs. However although MLVA cannot be expected to provide robust phylogenetic support, it offers a high level of discrimination as well as correct and unbiased clustering assignment. It is also currently the only simple and widely affordable technique which allows the study of large populations of samples: it is able to identify isolates which are only distantly related to known strains and clusters, in a more robust way than spoligotyping and consequently is appropriate to provide a rapid and global overview of a bacterial population. In the present study the MLVA clustering combined with spoligotyping allowed the clade assignment of all 435 isolates except for Percy556, which turned out to be a member of a previously unknown lineage. Similarly the two *M. africanum* isolates, placed in an outgroup position by MLVA clustering, proved to be defining new ancestral nodes within lineage 6. Percy717, an *M. africanum* West African 1 isolate from Gabon which was differentiated by MLVA from the other members of this sublineage is also defining a very ancestral node within lineage 5.

### Geographical origin of the different lineages

Phylogeographic studies have shown that lineages within *M. tuberculosis* strains can be associated with particular localisations in the world presumably as a result of human migrations. Whilst the Republic of Djibouti occupies a very small surface, the patients harbour strains of all the major MTBC clades specifically affecting humans. The larger group possess the deletion of spacers S33 to S36 the signature of lineage 4 isolates. MLVA distributes the H, T and X clades isolates into two subfamilies, lineage 4-H37 and lineage 4-CDC. The LAM spoligotypes mostly correspond to LAM1 and LAM9. Interestingly, among the lineage 4-CDC, a group of isolates shows a large deletion of spacer S4 to S23 previously unreported and likely to be a recently emerging clone in Djibouti (here called the Djibouti family).

Lineage 3 CAS represents the second largest clade and is separated into CAS-Kili previously found in Tanzania [55] and CAS-Delhi highly represented in India and Saudi Arabia [56]. The genetic diversity inside the CAS-Delhi family suggests a long time of evolution or recolonisation by more diverse sublineages whereas the CAS-Kili family appears to be emerging in the Horn of Africa, or has not had the opportunity to diversify into multiple geotypes. The EAI clade from lineage 1 displays the larger diversity. However, we observe no Manu1, 2 or 3 lineage isolate. The Manu1-3 lineage is relatively frequent in India where it has been shown to be related to the EAI clade and to belong to lineage 1 characterised by the RD239 deletion [49], [57], [58].

To compare the strain diversity in Djibouti to that of neighbouring countries and countries of East Africa mostly spoligotyping data are available in the literature as summarised in Table S6 [46], [55], [56], [59], [60], [61], [62], [63], [64], [65]. SITVITWEB also provides a very convenient tool to view available data. The clade identification from the spoligotype is not always straightforward, and only 70 to 80% of isolates have been assigned to a clade. In brief, the pattern in East Africa, Egypt and Saudi Arabia is highly similar, with a predominance of lineages 1, 3 and 4. In South Africa a study of 252 isolates showed the predominance of lineage 2 and lineage 4 strains [64]. In Ethiopia, most of the 141 isolates in SIVITWEB are lineage 4 (T, in particular T3-ETH). In Sudan, 2/3 of the 49 isolates investigated are CAS lineage 3.

In agreement with previous investigations, only two *M. africanum* and one *M. bovis* strains were found in the present study [43]. The *M. africanum* lineages 5 and 6 are currently mostly limited to West Africa where they are very abundant. They represent for instance an estimated 20% of MTBC isolates in Burkina-Faso [31] but are rare in East African countries [66]. Curiously the two lineage 6 *M. africanum* isolates from Djibouti appear to possess spacer S8 which is absent from West African isolates, and whole genome sequence analysis places them in a new branch defining a node closer to the junction with the *M. bovis* lineage.

### A new method for whole genome SNP identification

There is not yet a standardised approach to identify phylogenetically relevant SNPs in whole genome sequence data. Very often, SNPs deduced by aligning short reads, contigs, or whole genome sequences on a reference genome are filtered in order to mask different kinds of repeated elements [14], [52], [67], [68]. At least two reasons can be invoked to justify this masking step. Firstly short reads cannot confidently be used to reconstitute such elements, and consequently, analyses using a mixture of fully sequenced genomes and NGS short reads data should not take into account SNPs located in repeated elements. Secondly, intrachromosomal homologous recombination within such elements have been shown to exist in *M. tuberculosis* [69]. The use of genetic variations resulting from internal shuffling to infer phylogeny would require specific tools compared to the analysis of *de novo* SNPs inherited clonally. It is also common practice to discard SNPs occurring in very close proximity, typically less than 10 bp apart [52]. As a result different investigations of the same sequence data may produce slightly different lists of SNPs. In the present investigation, no filtering step was applied to the initially produced list of SNPs. This is due to the constitution of pseudo-short reads from the fully sequenced genomes previous to the mapping step on the reference genome. In this way, most if not all genetic variations potentially resulting from intrachromosomal exchanges between repeated elements are *de facto* erased.

### Identification of a new lineage and branch length

Lineage 7 identified here is an excellent candidate for representing a local *M. tuberculosis* geotype as predicted by evolutionary models for clonal bacterial species [42]. Percy256 and Percy556 with spoligotype SIT910 happen to share a remarkable characteristic in terms of spoligotype, the presence of spacers S34 and S39. Apart from the rare SIT523 Manu ancestor with all forty-three spacers identified predominantly in Asia, this signature is also present in SIT343, SIT415, SIT461, SIT1729 and SIT1764. SIT415 and SIT461 were observed in India and countries of Indian emigration. In contrast, SIT343 was observed in 5 isolates, two from Ethiopia, two from the UK, and one from the USA. SIT1729 was observed in one Somalian and one Ethiopian isolate. SIT1764 was observed in two Kenyan isolates. These isolates are candidates to represent additional geotypes within lineage 7. The observation that the few suspected members of lineage 7 (sharing the same or significantly similar spoligotype) as well as all *“M. canettii”* isolates with an unambiguous geographic origin originate from the Horn of Africa suggests that lineage 7 emerged and remained in this region of the world. Interestingly, 5 among 141 isolates from Ethiopia present in SITVIWEB contain both S34 and S39. This is much higher than the proportion observed in Djibouti, and may suggest that Ethiopia contains the epicentre of lineage 7, or at least is closer to it. SITVITWEB currently contains little or no data from additional neighboring countries such as Somalia or Uganda. In the present study thirty percent of the 411 isolates collected in Djibouti were from Ethiopian or Somalian patients so that we cannot exclude that Percy556 originate from Ethiopia via an Ethiopian patient. Due to the strict anonymisation of samples, this question could not be addressed here.

The topology of the MTBC phylogeny, and in particular the relative position of lineage 7, is a clear indication that all three superlineages constituting the extant MTBC emerged separately from the Horn of Africa. The first two superlineages, comprising lineage 1, and lineages 5, 6, Bovis respectively, might have emerged almost simultaneously. A more precise positioning of the root of the MTBC will be required in order to firmly establish the temporal order of emergence of these two superlineages. The third superlineage, comprising lineages 2, 3, and 4, emerged later. One striking observation is that the length from root to tip is very similar or even slightly longer in the new lineage 7 compared to the other lineages in spite of the fact that lineage 7 is much less widespread than others. This might be consistent with the observation by Ford et al. [54] that the mutation rate of *M. tuberculosis* is very similar during latency, active disease or even exponential growth in culture media. Hopefully, future investigations of ancient *M. tuberculosis* DNA by whole genome sequencing might allow the more precise calibration of the molecular clock within the MTBC. Under the 5,000–40,000 years current estimate, the third superlineage which later colonized the whole of Eurasia would have emerged ca 300–3000 years after the first two. In that respect, it may indeed be called the Modern superlineage, whereas the first two are the Ancient superlineages. Subsequent worldwide spread of these three independent branches via human migration resulted in the current MTBC geotypes. The animal lineage is the unique exception where host preference has been the basis for true ecotypes emergence and preservation [42]. It is remarkable that the Modern superlineage, which would have split 300–3000 years after the first two, was able to spread, suggesting that the area was at that time a major demographic and presumably economic actor. This may also suggest that it possessed a selective advantage and comparative genome analysis of lineage 7 versus lineage 2-3-4 may help identify the underlying genetic factors. It is also remarkable that no other similarly successful event occurred afterwards, suggesting that the human niche was by then already largely occupied by the descendants of the first three superlineages or that the region experienced major economical changes. Interestingly, in their investigation of Egyptian mummies, Zink et al. detected both the Ancient (*M. africanum*) and the Modern lineages. More precisely, all four samples from the oldest tomb investigated (2050–1650 BC) had the characteristic *M. africanum* spoligotype, whereas five out of six samples investigated in tombs dated 1450–500 or 1250–500 BC were Modern with a typical lineage 4 spoligotype signature (incidentally, 3 among the *M. africanum* isolates possessed spacer 8, as observed here for Percy34 and Percy117, which is a rare feature among contemporary *M. africanum*). It is tempting to speculate that within a few centuries, Modern superlineage representatives replaced the Ancient Africanum lineage in Egypt and East Africa.

The transience of *M. tuberculosis* geotypes and dynamics of *M. tuberculosis* population structure in a given place [70] is further illustrated in the present investigation. In the snapshot of the *M. tuberculosis* diversity in the republic of Djibouti, some lineages are clearly new comers, and the country may be a place of intense competition between the many geotypes which are present. The rare lineage 2 Beijing isolates identified in the present study are most likely the result of re-import events, because no member of the RD150-, RD181- or RD142-intact sub-lineages were observed. Similarly, it will be interesting in future investigations to characterise in more details lineage 1 EAI and lineage 3 CAS isolates from the Horn of Africa, in order to better understand at what time the different sublineages were re-introduced. Some of the isolates investigated here are positioned on long MLVA branches and may be relevant choices for future whole genome SNP analysis.

## Materials and Methods

### Ethics statement

The study obtained approval of local ethics committee (the Paul Faure Centre Ethics Committee). The committee estimated that patient informed consent was not necessary since collection of sputum or pus was part of the patients' usual care and no additional sample was necessary for the present study. All samples were anonymised.

#### Patients

In Djibouti, Republic of Djibouti, Horn of Africa, the Paul Faure Anti-tuberculosis Centre (PFC) cares for patients with suspected TB. Among 421 cultures positive for mycobacteria obtained between 1997 and 2011, 411 were shown to contain a unique MTBC genotype and were retained for the present study. The origin of the patients was not always known but about 70% of them were Djiboutian, and the others were from Somalia or Ethiopia. Sputum was collected and sent to the Percy military hospital, France, for isolation of *M. tuberculosis*. In addition 24 isolates recovered from patients in neighbouring countries were included in the study: Somalia (2 isolates), Sudan (19 isolate) and Kenya (3 isolates). For comparison, two “*M. canettii*” isolates, Percy525 and Percy673 [13], and the reference strains H37Rv and CDC1551 were included.

#### Sample processing and identification tests

Acid-fast bacilli (AFB) in samples were detected by auramine staining. Positive results were confirmed by Ziehl-Neelsen staining. Lowenstein-Jensen (LJ) slants and Coletsos slants were inoculated with each sample. The samples were cultured in Mycobacterial Growth Indicator Tubes (MGIT, Becton-Dickinson, Le Pont de Claix, France) and MTBC were directly detected by 16S rRNA amplification (Amplified *Mycobacterium tuberculosis* Direct Test, AMTD; Gen-Probe-bioMerieux, Lyon, France). Mycobacteria were identified according to conventional biochemical procedures. The concentration of thiophen-2-carboxylic acid hydrazide used was 2 g/L. Each positive culture was also tested with an *M. tuberculosis* complex DNA probe (Accuprobe; Gen-Probe-bioMerieux, Lyon, France). Susceptibility of the MTBC strains to isoniazid (INH, 0.2 g/L), rifampicin (RIF, 40 g/L), streptomycin (SM, 4 g/L), ethambutol (EMB, 2 g/L) were determined by the proportion method on LJ medium.

### DNA preparation

Thermolysates were prepared by suspending bacteria into water and heating at 90°C for 30 min. DNA for whole genome draft sequencing was prepared as follows. Bacteria were killed by heating 20 min at 80°C and treated by lysozyme at 2 mg/ml final concentration for 30 min at room temperature. Then one volume of lysis buffer (20 mM Tris PH 8, 20 mM EDTA PH 8, 20 mM NaCl, 1%SDS) and 50 µg/mL Proteinase K were added and the lysate was incubated 2 hours at 50°C. NaCl was added to a final concentration of 0.7 M, followed by CTAB at a 1% final concentration and the solution was thoroughly mixed before incubation for 10 min at 65°C. Chloroform extraction was performed and the aqueous phase containing DNA was purified using two successive extractions with phenol and chloroform. Finally the DNA was precipitated with 2 volumes ethanol.

### MLVA data management and analyses

PCR amplification of VNTR loci from thermolysates and electrophoresis of products on agarose gels were as described in [48]. The twenty-four loci selection previously proposed by Fabre et al. was used (Table S2; [13]) with the list of primers indicated in Table S7. The reference H37Rv *M. tuberculosis* strain was included as a control in the MLVA analysis. The MLVA typing data is presented in Table S8 and has been incorporated in the *Mycobacterium tuberculosis* database on the MLVAbank website [71].

Gel images were analyzed using the BioNumerics software package version 6.6 (Applied-Maths, Sint-Martens-Latem, Belgium) as previously described [48]. The number of repeats in each allele was deduced from the amplicon size. The resulting data were analyzed as a character dataset. Clustering analysis was done using the categorical parameter and the UPGMA coefficient. The minimum spanning tree was constructed with the following options: (a) in case of equivalent solutions in terms of calculated distances, the selected tree was the one containing the highest number of links between genotypes differing at only one locus (“Highest number of single locus variants” option); (b) the creation of hypothetical types (missing links) reducing the total length of the tree was allowed.

### Analysis of the DR locus by Spoligotyping and PCR amplification

Spoligotyping was performed using a membrane from Isogen (De Meern, The Netherlands) as described by the manufacturer. The resulting data was imported into BioNumerics as a character data set. Spoligotypes were analysed using SITVITWEB [44], [57], [72].

### Deletion analysis

The presence/absence of the RD7, RD9 and Tbd1 regions was analysed by PCR using the primers described by Brosch et al. [10]. Beijing-family strains were analysed for the RD142, RD150 and RD181 deletion as described [73]. Investigation of newly observed regions of deletion in strain Percy556 was performed using primers localised inside the region and in flanking sequences. For Del79 the internal primers were MCE3F-For 5′-ATATCCCGTCGGACCTCAAG-3′ and MCE3F-Rev 5′-TGGGTCTGCGAATCCAGCAC-3′ and the flanking primers were P556Del79-For 5′-GGCTGATCGGGCAGGTGATC-3′ and P556Del79-Rev 5′-CATTACGCTCACAACTCGCA-3′. For del154 the internal primers were RmlB-For 5′-ACGGCGAGCGCGGGAGTTAA-3′ and RmlB-Rev 5′-TGCCCGATTCTTCGTCGAGG-3′.

### Draft whole genome sequencing and data analysis

Purified DNA from the seven isolates Percy34, Percy119, Percy209, Percy256, Percy556, Percy 644 and Percy717 was used for paired-end sequencing on the Illumina HiSeq2000 platform (BaseClear, The Netherland, or Imagif, Gif sur Yvette, France). Complete genome sequences from *M. bovis* AF2122/97 (NC_002945.3), BCG Pasteur 1173P2 (NC_008769.1), BCG Tokyo 172 (NC_012207.1), *M. tuberculosis* H37Rv (NC_000962.2), CDC1551 (NC_002755.2), F11 (NC_009565.1), H37Ra (NC_009525.1), KZN605 (NC_018078.1), KZN 1435 (NC_012943.1), KZN4207 (NC_016768.1), CCDC5180 (NC_017522.1), CTRI-2 (NC_017524.1), Erdman (AP012340.1), “*M. canettii*” CIPT140010059 (NC_015848.1) were downloaded from Genbank. Sequence read archives were downloaded from the short reads archive (sra) hosted by NCBI [14], [52], [74] or from the *Mycobacterium tuberculosis* Comparative Sequencing and *Mycobacterium tuberculosis* Diversity Sequencing Projects, Broad Institute of Harvard and MIT [75].

In order to harmonize the SNP detection procedure, the fourteen whole-genome sequences were split into short overlapping 75 bp reads resulting in a 75× coverage. Artificial and real short reads data sets were mapped on the reference H37Rv strain genome using BioNumerics asking for a similarity of at least 90%. A set of SNPs with a coverage higher than 10× was deduced for each genome sequence data. Individual lists were compiled, removing SNP positions at which one or more isolate displayed an ambiguous residue call or missing data. The list of SNP positions and main characteristics is provided in Table S3 and the SNP data for each genome is provided in Table S4.

A minimum spanning tree was drawn using BioNumerics and allowing the creation of hypothetical intermediates. Nodes were numbered by BioNumerics and the list of SNPs contributing to each branch were automatically retrieved to be included in Table S3.

In order to identify missing regions in the genome of strain Percy 556 compared to H37Rv, a mapping assembly on this reference strain genome was done using BioNumerics. Missing regions larger than 2 kb and specific for Percy556 are listed in Table S5.

All NGS sequencing reads produced in the course of this work are deposited in the European Nucleotide Archive (ENA project accession ERP001885, [76]) maintained by the European Bioinformatics Institute (EBI).

In order to study the Interrupted Coding Sequences (ICDSs) identified by Deshayes et al. [51], a *de novo* assembly was carried out on the Percy256 sequence data. The assembly was performed on 3 millions Illumina 100 bp reads, using the BioNumerics assembly module (Power Assembler). The algorithm chosen for this step was Velvet, with k set to 31 and a minimum contig length of 1000 bp. The region corresponding to each ICDS was extracted from the H37Rv genome, and compared to the Percy256 *de novo* assembly.

## Supporting Information

Figure S1 **Dendrogram of the 435 isolates based upon MLVA24_Orsay_.** The spoligotype, strain Id, geographic origin, spoligotype clade, year of isolation and antibiotic resistance status are indicated. The color code used is as indicated in Figure 1. The dendrogram is based upon data produced using the previously published MLVA24_Orsay_ assay, the categorical coefficient and UPGMA clustering [13]. Two reference genomes (H37Rv and CDC1551) and two *M. canettii* isolates, Percy525 and Percy673 are included for comparison.
(PPT)

Table S1 Eighty spoligotypes observed in the isolate collection, and their frequency.
(XLS)

Table S2 MLVA panels associated with internet accessible databases.
(DOC)

Table S3 List of 13358 SNP positions. For each SNP the branch(es) in which they occurred is indicated. Nodes numbering is as indicated in Figure 7. When an SNP occurs in a region of overlap between two annotated open reading frames, both amino acid changes are indicated.
(XLS)

Table S4 SNP status in the different genomes.
(XLS)

Tables S5 List of the 13 deletions larger than 2 kb in Percy556 versus H37Rv.
(XLS)

Table S6 Studies of *M. tuberculosis* diversity in Eastern Africa countries.
(DOC)

Table S7 MLVA primers used for the MLVA24_Orsay_ assay.
(XLS)

Table S8 MLVA typing data.
(XLS)

## References

[1] Rothschild BM, Martin LD, Lev G, Bercovier H, Bar-Gal GK, et al (2001) *Mycobacterium tuberculosis* complex DNA from an extinct bison dated 17,000 years before the present. Clin Infect Dis 33: 305–311. 11438894 10.1086/321886

[2] Hershkovitz I, Donoghue HD, Minnikin DE, Besra GS, Lee OY, et al (2008) Detection and molecular characterization of 9,000-year-old *Mycobacterium tuberculosis* from a Neolithic settlement in the Eastern Mediterranean. PLoS One 3: e3426. 18923677 10.1371/journal.pone.0003426 PMC2565837

[3] Crubezy E, Ludes B, Poveda JD, Clayton J, Crouau-Roy B, et al (1998) Identification of *Mycobacterium* DNA in an Egyptian Pott's disease of 5,400 years old. C R Acad Sci III 321: 941–951. 9879471 10.1016/s0764-4469(99)80009-2

[4] Salo WL, Aufderheide AC, Buikstra J, Holcomb TA (1994) Identification of *Mycobacterium tuberculosis* DNA in a pre-Columbian Peruvian mummy. Proc Natl Acad Sci U S A 91: 2091–2094. 8134354 10.1073/pnas.91.6.2091 PMC43315

[5] Zink AR, Sola C, Reischl U, Grabner W, Rastogi N, et al (2003) Characterization of *Mycobacterium tuberculosis* complex DNAs from Egyptian mummies by spoligotyping. J Clin Microbiol 41: 359–367. 12517873 10.1128/JCM.41.1.359-367.2003 PMC149558

[6] Nerlich AG, Haas CJ, Zink A, Szeimies U, Hagedorn HG (1997) Molecular evidence for tuberculosis in an ancient Egyptian mummy. Lancet 350: 1404. 10.1016/S0140-6736(05)65185-9 9365482

[7] Taylor GM, Goyal M, Legge AJ, Shaw RJ, Young D (1999) Genotypic analysis of *Mycobacterium tuberculosis* from medieval human remains. Microbiology 145 Pt 4: 899–904. 10220169 10.1099/13500872-145-4-899

[8] Mays S, Taylor GM, Legge AJ, Young DB, Turner-Walker G (2001) Paleopathological and biomolecular study of tuberculosis in a medieval skeletal collection from England. Am J Phys Anthropol 114: 298–311. 11275959 10.1002/ajpa.1042

[9] Wilbur AK, Bouwman AS, Stone AC, Roberts CA, Pfister LA, et al (2009) Deficiencies and challenges in the study of ancient tuberculosis DNA. Journal of Archaeological Science 36: 1990–1997.

[10] Brosch R, Gordon SV, Marmiesse M, Brodin P, Buchrieser C, et al (2002) A new evolutionary scenario for the *Mycobacterium tuberculosis* complex. Proc Natl Acad Sci U S A 99: 3684–3689. 11891304 10.1073/pnas.052548299 PMC122584

[11] Fabre M, Koeck JL, Le Fleche P, Simon F, Herve V, et al (2004) High genetic diversity revealed by variable-number tandem repeat genotyping and analysis of hsp65 gene polymorphism in a large collection of “*Mycobacterium canettii*” strains indicates that the *M. tuberculosis* complex is a recently emerged clone of “*M. canettii*”. J Clin Microbiol 42: 3248–3255. 15243089 10.1128/JCM.42.7.3248-3255.2004 PMC446256

[12] Gutierrez MC, Brisse S, Brosch R, Fabre M, Omais B, et al (2005) Ancient origin and gene mosaicism of the progenitor of *Mycobacterium tuberculosis* . PLoS Pathog 1: e5. 16201017 10.1371/journal.ppat.0010005 PMC1238740

[13] Fabre M, Hauck Y, Soler C, Koeck JL, van Ingen J, et al (2010) Molecular characteristics of “*Mycobacterium canettii*” the smooth *Mycobacterium tuberculosis* bacilli. Infect Genet Evol 10: 1165–1173. 20692377 10.1016/j.meegid.2010.07.016

[14] Comas I, Chakravartti J, Small PM, Galagan J, Niemann S, et al (2010) Human T cell epitopes of *Mycobacterium tuberculosis* are evolutionarily hyperconserved. Nat Genet 42: 498–503. 20495566 10.1038/ng.590 PMC2883744

[15] Smith NH, Hewinson RG, Kremer K, Brosch R, Gordon SV (2009) Myths and misconceptions: the origin and evolution of *Mycobacterium tuberculosis* . Nat Rev Microbiol 7: 537–544. 19483712 10.1038/nrmicro2165

[16] Veyrier FJ, Dufort A, Behr MA (2011) The rise and fall of the *Mycobacterium tuberculosis* genome. Trends Microbiol 19: 156–161. 21277778 10.1016/j.tim.2010.12.008

[17] Koeck JL, Fabre M, Simon F, Daffe M, Garnotel E, et al (2010) Clinical characteristics of the smooth tubercle bacilli ‘*Mycobacterium canettii*’ infection suggest the existence of an environmental reservoir. Clin Microbiol Infect 10.1111/j.1469-0691.2010.03347.x 20831613

[18] Smith NH, Kremer K, Inwald J, Dale J, Driscoll JR, et al (2006) Ecotypes of the *Mycobacterium tuberculosis* complex. J Theor Biol 239: 220–225. 16242724 10.1016/j.jtbi.2005.08.036

[19] Namouchi A, Didelot X, Schock U, Gicquel B, Rocha EP (2012) After the bottleneck: Genome-wide diversification of the *Mycobacterium tuberculosis* complex by mutation, recombination, and natural selection. Genome Res 10.1101/gr.129544.111 PMC3317154 22377718

[20] Kamerbeek J, Schouls L, Kolk A, van Agterveld M, van Soolingen D, et al (1997) Simultaneous detection and strain differentiation of *Mycobacterium tuberculosis* for diagnosis and epidemiology. J Clin Microbiol 35: 907–914. 9157152 10.1128/jcm.35.4.907-914.1997 PMC229700

[21] Kremer K, van Soolingen D, Frothingham R, Haas WH, Hermans PW, et al (1999) Comparison of methods based on different molecular epidemiological markers for typing of *Mycobacterium tuberculosis* complex strains: interlaboratory study of discriminatory power and reproducibility. J Clin Microbiol 37: 2607–2618. 10405410 10.1128/jcm.37.8.2607-2618.1999 PMC85295

[22] Supply P, Allix C, Lesjean S, Cardoso-Oelemann M, Rusch-Gerdes S, et al (2006) Proposal for standardization of optimized mycobacterial interspersed repetitive unit-variable-number tandem repeat typing of *Mycobacterium tuberculosis* . J Clin Microbiol 44: 4498–4510. 17005759 10.1128/JCM.01392-06 PMC1698431

[23] Filliol I, Motiwala AS, Cavatore M, Qi W, Hernando Hazbon M, et al (2006) Global Phylogeny of *Mycobacterium tuberculosis* Based on Single Nucleotide Polymorphism (SNP) Analysis: Insights into Tuberculosis Evolution, Phylogenetic Accuracy of Other DNA Fingerprinting Systems, and Recommendations for a Minimal Standard SNP Set. J Bacteriol 188: 759–772. 16385065 10.1128/JB.188.2.759-772.2006 PMC1347298

[24] Hershberg R, Lipatov M, Small PM, Sheffer H, Niemann S, et al (2008) High functional diversity in *Mycobacterium tuberculosis* driven by genetic drift and human demography. PLoS Biol 6: e311. 19090620 10.1371/journal.pbio.0060311 PMC2602723

[25] Comas I, Homolka S, Niemann S, Gagneux S (2009) Genotyping of genetically monomorphic bacteria: DNA sequencing in *Mycobacterium tuberculosis* highlights the limitations of current methodologies. PLoS One 4: e7815. 19915672 10.1371/journal.pone.0007815 PMC2772813

[26] Marmiesse M, Brodin P, Buchrieser C, Gutierrez C, Simoes N, et al (2004) Macro-array and bioinformatic analyses reveal mycobacterial ‘core’ genes, variation in the ESAT-6 gene family and new phylogenetic markers for the *Mycobacterium tuberculosis* complex. Microbiology 150: 483–496. 14766927 10.1099/mic.0.26662-0

[27] Smith NH, Upton P (2011) Naming spoligotype patterns for the RD9-deleted lineage of the *Mycobacterium tuberculosis* complex; Infect Genet Evol www.Mbovis.org 10.1016/j.meegid.2011.08.002 21855653

[28] Shabbeer A, Cowan LS, Ozcaglar C, Rastogi N, Vandenberg SL, et al (2012) TB-Lineage: An online tool for classification and analysis of strains of *Mycobacterium tuberculosis* complex. Infect Genet Evol 12: 789–797. 22406225 10.1016/j.meegid.2012.02.010

[29] Ledru S, Cauchoix B, Yameogo M, Zoubga A, Lamande-Chiron J, et al (1996) Impact of short-course therapy on tuberculosis drug resistance in South-West Burkina Faso. Tuber Lung Dis 77: 429–436. 8959147 10.1016/s0962-8479(96)90116-1

[30] Intemann CD, Thye T, Niemann S, Browne EN, Amanua Chinbuah M, et al (2009) Autophagy gene variant IRGM -261T contributes to protection from tuberculosis caused by *Mycobacterium tuberculosis* but not by *M. africanum* strains. PLoS Pathog 5: e1000577. 19750224 10.1371/journal.ppat.1000577 PMC2735778

[31] Gomgnimbou MK, Refregier G, Diagbouga SP, Adama S, Kabore A, et al (2012) Spoligotyping of *Mycobacterium africanum*, Burkina Faso. Emerg Infect Dis 18: 117–119. 22257494 10.3201/eid1801.110275 PMC3310091

[32] Bentley SD, Comas I, Bryant JM, Walker D, Smith NH, et al (2012) The Genome of *Mycobacterium Africanum* West African 2 Reveals a Lineage-Specific Locus and Genome Erosion Common to the *M. tuberculosis* Complex. PLoS Negl Trop Dis 6: e1552. 22389744 10.1371/journal.pntd.0001552 PMC3289620

[33] van Ingen J, Rahim Z, Mulder A, Boeree MJ, Simeone R, et al (2012) Characterization of *Mycobacterium orygis* as *M. tuberculosis* Complex Subspecies. Emerg Infect Dis 18: 653–655. 22469053 10.3201/eid1804.110888 PMC3309669

[34] Kapur V, Whittam TS, Musser JM (1994) Is *Mycobacterium tuberculosis* 15,000 years old? J Infect Dis 170: 1348–1349. 7963745 10.1093/infdis/170.5.1348

[35] Sreevatsan S, Pan X, Stockbauer KE, Connell ND, Kreiswirth BN, et al (1997) Restricted structural gene polymorphism in the *Mycobacterium tuberculosis* complex indicates evolutionarily recent global dissemination. Proc Natl Acad Sci U S A 94: 9869–9874. 9275218 10.1073/pnas.94.18.9869 PMC23284

[36] Wirth T, Hildebrand F, Allix-Beguec C, Wolbeling F, Kubica T, et al (2008) Origin, spread and demography of the *Mycobacterium tuberculosis* complex. PLoS Pathog 4: e1000160. 18802459 10.1371/journal.ppat.1000160 PMC2528947

[37] Fagundes NJ, Ray N, Beaumont M, Neuenschwander S, Salzano FM, et al (2007) Statistical evaluation of alternative models of human evolution. Proc Natl Acad Sci U S A 104: 17614–17619. 17978179 10.1073/pnas.0708280104 PMC2077041

[38] Li JZ, Absher DM, Tang H, Southwick AM, Casto AM, et al (2008) Worldwide human relationships inferred from genome-wide patterns of variation. Science 319: 1100–1104. 18292342 10.1126/science.1153717

[39] Schlebusch CM, Skoglund P, Sjodin P, Gattepaille LM, Hernandez D, et al (2012) Genomic variation in seven Khoe-San groups reveals adaptation and complex African history. Science 338: 374–379. 22997136 10.1126/science.1227721 PMC8978294

[40] Mellars P (2006) Going east: new genetic and archaeological perspectives on the modern human colonization of Eurasia. Science 313: 796–800. 16902130 10.1126/science.1128402

[41] Cohan FM (2002) What are bacterial species? Annu Rev Microbiol 56: 457–487. 12142474 10.1146/annurev.micro.56.012302.160634

[42] Cohan FM, Koeppel AF (2008) The origins of ecological diversity in prokaryotes. Curr Biol 18: R1024–1034. 19000803 10.1016/j.cub.2008.09.014

[43] Godreuil S, Renaud F, Choisy M, Depina JJ, Garnotel E, et al (2009) Highly structured genetic diversity of the *Mycobacterium tuberculosis* population in Djibouti. Clin Microbiol Infect 10.1111/j.1469-0691.2009.03025.x 19694762

[44] Demay C, Liens B, Burguiere T, Hill V, Couvin D, et al (2012) SITVITWEB - A publicly available international multimarker database for studying *Mycobacterium tuberculosis* genetic diversity and molecular epidemiology. Infect Genet Evol 12: 755–766. 22365971 10.1016/j.meegid.2012.02.004

[45] Allix-Beguec C, Harmsen D, Weniger T, Supply P, Niemann S (2008) Evaluation and strategy for use of MIRU-VNTRplus, a multifunctional database for online analysis of genotyping data and phylogenetic identification of *Mycobacterium tuberculosis* complex isolates. J Clin Microbiol 46: 2692–2699. 18550737 10.1128/JCM.00540-08 PMC2519508

[46] Helal ZH, Ashour MS, Eissa SA, Abd-Elatef G, Zozio T, et al (2009) Unexpectedly high proportion of ancestral Manu genotype *Mycobacterium tuberculosis* strains cultured from tuberculosis patients in Egypt. J Clin Microbiol 47: 2794–2801. 19553569 10.1128/JCM.00360-09 PMC2738058

[47] Viana-Niero C, Gutierrez C, Sola C, Filliol I, Boulahbal F, et al (2001) Genetic diversity of *Mycobacterium africanum* clinical isolates based on IS6110-restriction fragment length polymorphism analysis, spoligotyping, and variable number of tandem DNA repeats. J Clin Microbiol 39: 57–65. 11136749 10.1128/JCM.39.1.57-65.2001 PMC87680

[48] Le Flèche P, Fabre M, Denoeud F, Koeck JL, Vergnaud G (2002) High resolution, on-line identification of strains from the *Mycobacterium tuberculosis* complex based on tandem repeat typing. BMC Microbiol 2: 37. 12456266 10.1186/1471-2180-2-37 PMC140014

[49] Thomas SK, Iravatham CC, Moni BH, Kumar A, Archana BV, et al (2011) Modern and ancestral genotypes of *Mycobacterium tuberculosis* from Andhra Pradesh, India. PLoS One 6: e27584. 22114678 10.1371/journal.pone.0027584 PMC3219672

[50] Wan K, Liu J, Hauck Y, Zhang Y, Zhao X, et al (2011) Investigation on *Mycobacterium tuberculosis* diversity in China and the origin of the Beijing clade. PLoS One 6: e29190. 22220207 10.1371/journal.pone.0029190 PMC3248407

[51] Deshayes C, Perrodou E, Euphrasie D, Frapy E, Poch O, et al (2008) Detecting the molecular scars of evolution in the *Mycobacterium tuberculosis* complex by analyzing interrupted coding sequences. BMC Evol Biol 8: 78. 18325090 10.1186/1471-2148-8-78 PMC2277376

[52] Gardy JL, Johnston JC, Ho Sui SJ, Cook VJ, Shah L, et al (2011) Whole-genome sequencing and social-network analysis of a tuberculosis outbreak. N Engl J Med 364: 730–739. 21345102 10.1056/NEJMoa1003176

[53] Zumarraga M, Bigi F, Alito A, Romano MI, Cataldi A (1999) A 12.7 kb fragment of the *Mycobacterium tuberculosis* genome is not present in *Mycobacterium bovis* . Microbiology 145 Pt 4: 893–897. 10220168 10.1099/13500872-145-4-893

[54] Ford CB, Lin PL, Chase MR, Shah RR, Iartchouk O, et al (2011) Use of whole genome sequencing to estimate the mutation rate of *Mycobacterium tuberculosis* during latent infection. Nat Genet 43: 482–486. 21516081 10.1038/ng.811 PMC3101871

[55] Kibiki GS, Mulder B, Dolmans WM, de Beer JL, Boeree M, et al (2007) *M. tuberculosis* genotypic diversity and drug susceptibility pattern in HIV-infected and non-HIV-infected patients in northern Tanzania. BMC Microbiol 7: 51. 17540031 10.1186/1471-2180-7-51 PMC1913919

[56] Al-Hajoj SA, Zozio T, Al-Rabiah F, Mohammad V, Al-Nasser M, et al (2007) First insight into the population structure of *Mycobacterium tuberculosis* in Saudi Arabia. J Clin Microbiol 45: 2467–2473. 17507515 10.1128/JCM.02293-06 PMC1951255

[57] Brudey K, Driscoll JR, Rigouts L, Prodinger WM, Gori A, et al (2006) *Mycobacterium tuberculosis* complex genetic diversity: mining the fourth international spoligotyping database (SpolDB4) for classification, population genetics and epidemiology. BMC Microbiol 6: 23. 16519816 10.1186/1471-2180-6-23 PMC1468417

[58] Flores L, Van T, Narayanan S, Deriemer K, Kato-Maeda M, et al (2007) Large Sequence Polymorphisms Classify *Mycobacterium tuberculosis* Strains with Ancestral Spoligotyping Patterns. J Clin Microbiol 45: 3393–3395. 17699643 10.1128/JCM.00828-07 PMC2045339

[59] Asiimwe BB, Ghebremichael S, Kallenius G, Koivula T, Joloba ML (2008) *Mycobacterium tuberculosis* spoligotypes and drug susceptibility pattern of isolates from tuberculosis patients in peri-urban Kampala, Uganda. BMC Infect Dis 8: 101. 18662405 10.1186/1471-2334-8-101 PMC2519071

[60] Eldholm V, Matee M, Mfinanga SG, Heun M, Dahle UR (2006) A first insight into the genetic diversity of *Mycobacterium tuberculosis* in Dar es Salaam, Tanzania, assessed by spoligotyping. BMC Microbiol 6: 76. 16970826 10.1186/1471-2180-6-76 PMC1592105

[61] Ferdinand S, Sola C, Chanteau S, Ramarokoto H, Rasolonavalona T, et al (2005) A study of spoligotyping-defined *Mycobacterium tuberculosis* clades in relation to the origin of peopling and the demographic history in Madagascar. Infect Genet Evol 5: 340–348. 16168940 10.1016/j.meegid.2004.10.002

[62] Mulenga C, Shamputa IC, Mwakazanga D, Kapata N, Portaels F, et al (2010) Diversity of *Mycobacterium tuberculosis* genotypes circulating in Ndola, Zambia. BMC Infect Dis 10: 177. 20565802 10.1186/1471-2334-10-177 PMC2906459

[63] Viegas SO, Machado A, Groenheit R, Ghebremichael S, Pennhag A, et al (2010) Molecular diversity of *Mycobacterium tuberculosis* isolates from patients with pulmonary tuberculosis in Mozambique. BMC Microbiol 10: 195. 20663126 10.1186/1471-2180-10-195 PMC2914001

[64] Stavrum R, Mphahlele M, Ovreas K, Muthivhi T, Fourie PB, et al (2009) High diversity of *Mycobacterium tuberculosis* genotypes in South Africa and preponderance of mixed infections among ST53 isolates. J Clin Microbiol 47: 1848–1856. 19386854 10.1128/JCM.02167-08 PMC2691085

[65] Sharaf Eldin GS, Fadl-Elmula I, Ali MS, Ali AB, Salih AL, et al (2011) Tuberculosis in Sudan: a study of *Mycobacterium tuberculosis* strain genotype and susceptibility to anti-tuberculosis drugs. BMC Infect Dis 11: 219. 21846389 10.1186/1471-2334-11-219 PMC3166935

[66] de Jong BC, Antonio M, Awine T, Ogungbemi K, de Jong YP, et al (2009) Use of spoligotyping and large sequence polymorphisms to study the population structure of the *Mycobacterium tuberculosis* complex in a cohort study of consecutive smear-positive tuberculosis cases in The Gambia. J Clin Microbiol 47: 994–1001. 19193842 10.1128/JCM.01216-08 PMC2668362

[67] Weiner B, Gomez J, Victor TC, Warren RM, Sloutsky A, et al (2012) Independent large scale duplications in multiple *M. tuberculosis* lineages overlapping the same genomic region. PLoS One 7: e26038. 22347359 10.1371/journal.pone.0026038 PMC3274525

[68] Ford C, Yusim K, Ioerger T, Feng S, Chase M, et al (2012) *Mycobacterium tuberculosis* - Heterogeneity revealed through whole genome sequencing. Tuberculosis (Edinb) 92: 194–201. 22218163 10.1016/j.tube.2011.11.003 PMC3323677

[69] Uplekar S, Heym B, Friocourt V, Rougemont J, Cole ST (2011) Comparative genomics of *esx* genes from clinical isolates of *Mycobacterium tuberculosis* provides evidence for gene conversion and epitope variation. Infect Immun 79: 4042–4049. 21807910 10.1128/IAI.05344-11 PMC3187259

[70] Buu TN, Huyen MN, Lan NT, Quy HT, Hen NV, et al (2009) The Beijing genotype is associated with young age and multidrug-resistant tuberculosis in rural Vietnam. Int J Tuberc Lung Dis 13: 900–906. 19555542

[71] MLVAbank website. Available: http://mlva.u-psud.fr. Accessed 2012 Nov 29.

[72] SITVITWEB. Available: http://www.pasteur-guadeloupe.fr:8081/SITVIT_ONLINE/. Accessed 2012 Nov 29.

[73] Tsolaki AG, Gagneux S, Pym AS, Goguet de la Salmoniere YO, Kreiswirth BN, et al (2005) Genomic deletions classify the Beijing/W strains as a distinct genetic lineage of *Mycobacterium tuberculosis* . J Clin Microbiol 43: 3185–3191. 16000433 10.1128/JCM.43.7.3185-3191.2005 PMC1169157

[74] NCBI Sequence Read Archive. Available: http://www.ncbi.nlm.nih.gov/sra. Accessed 2012 Nov 29.

[75] The Broad institute website. Available: http://www.broadinstitute.org/. Accessed 2012 Nov 29.

[76] ENA project accession ERP001885. Available: http://www.ebi.ac.uk/ena/data/view/ERP001885. Accessed 2012 Nov 29.

