# Supplementary figures and images for: Significance of the Identification in the Horn of Africa of an Exceptionally Deep Branching Mycobacterium tuberculosis Clade
Source: PLoS One. 2012 Dec 27;7(12):e52841. doi: 10.1371/journal.pone.0052841 (PMC3531362; doi:10.1371/journal.pone.0052841)

## Slide 1
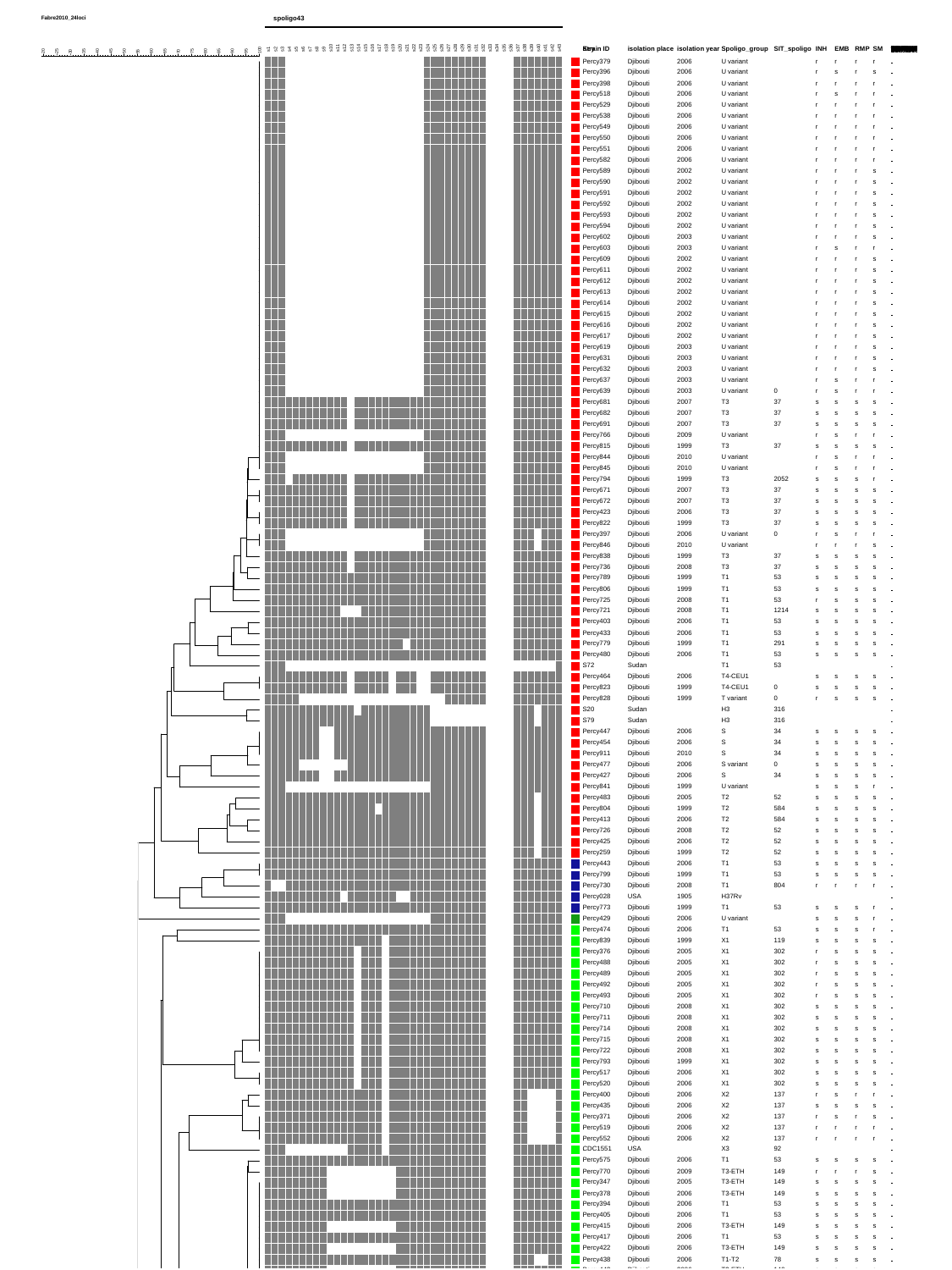

## Slide 2
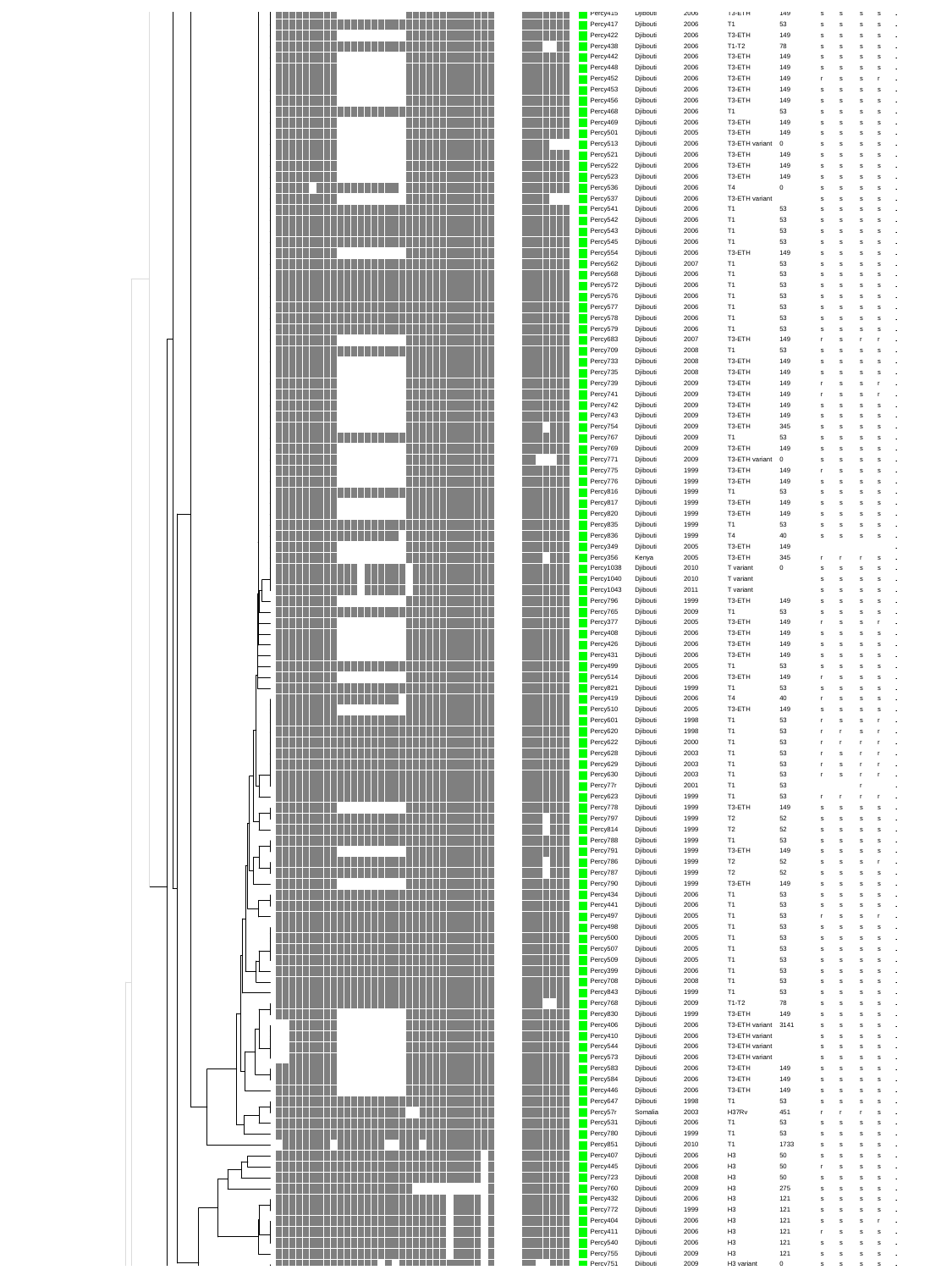

## Slide 3
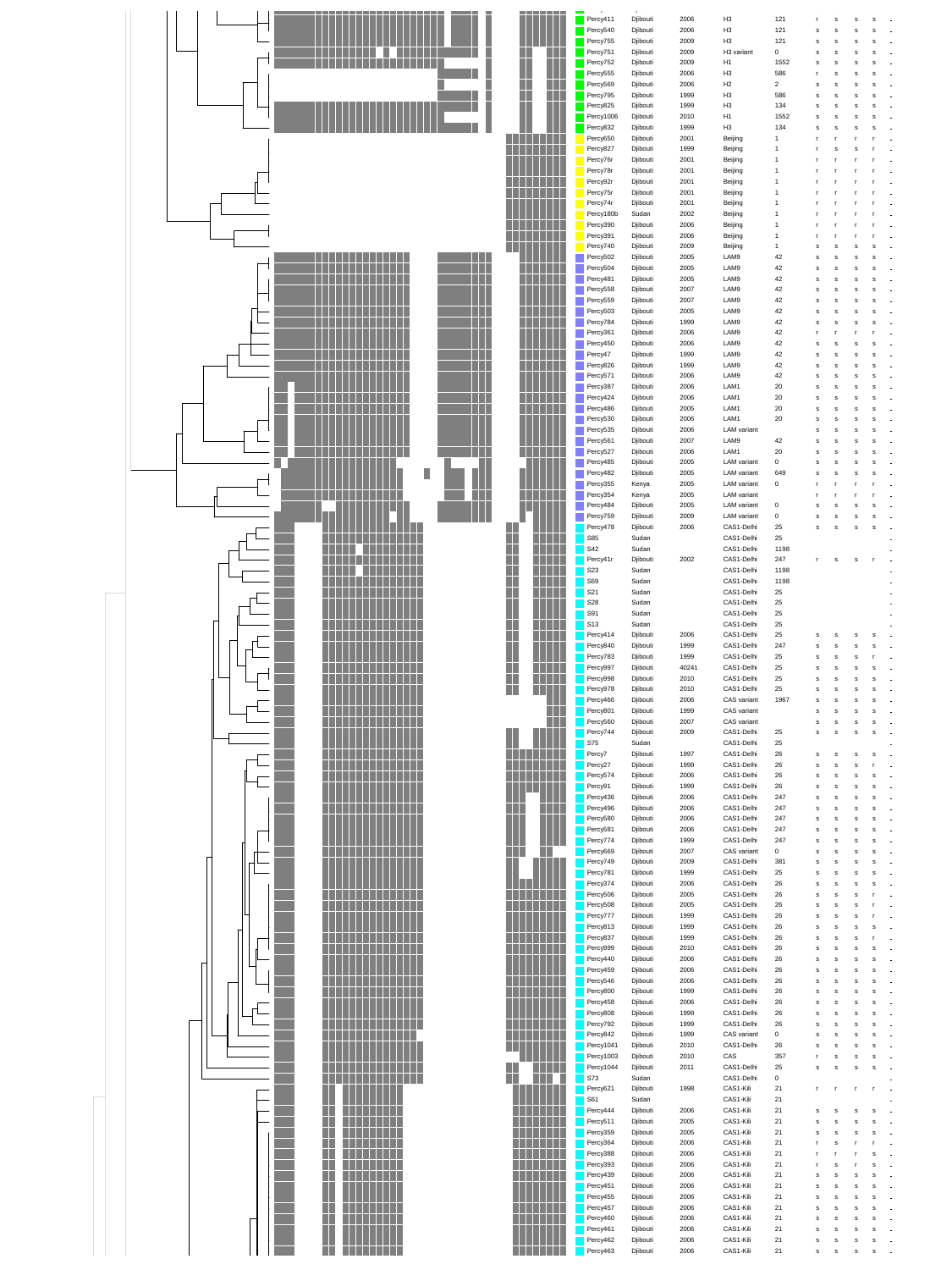

## Slide 4
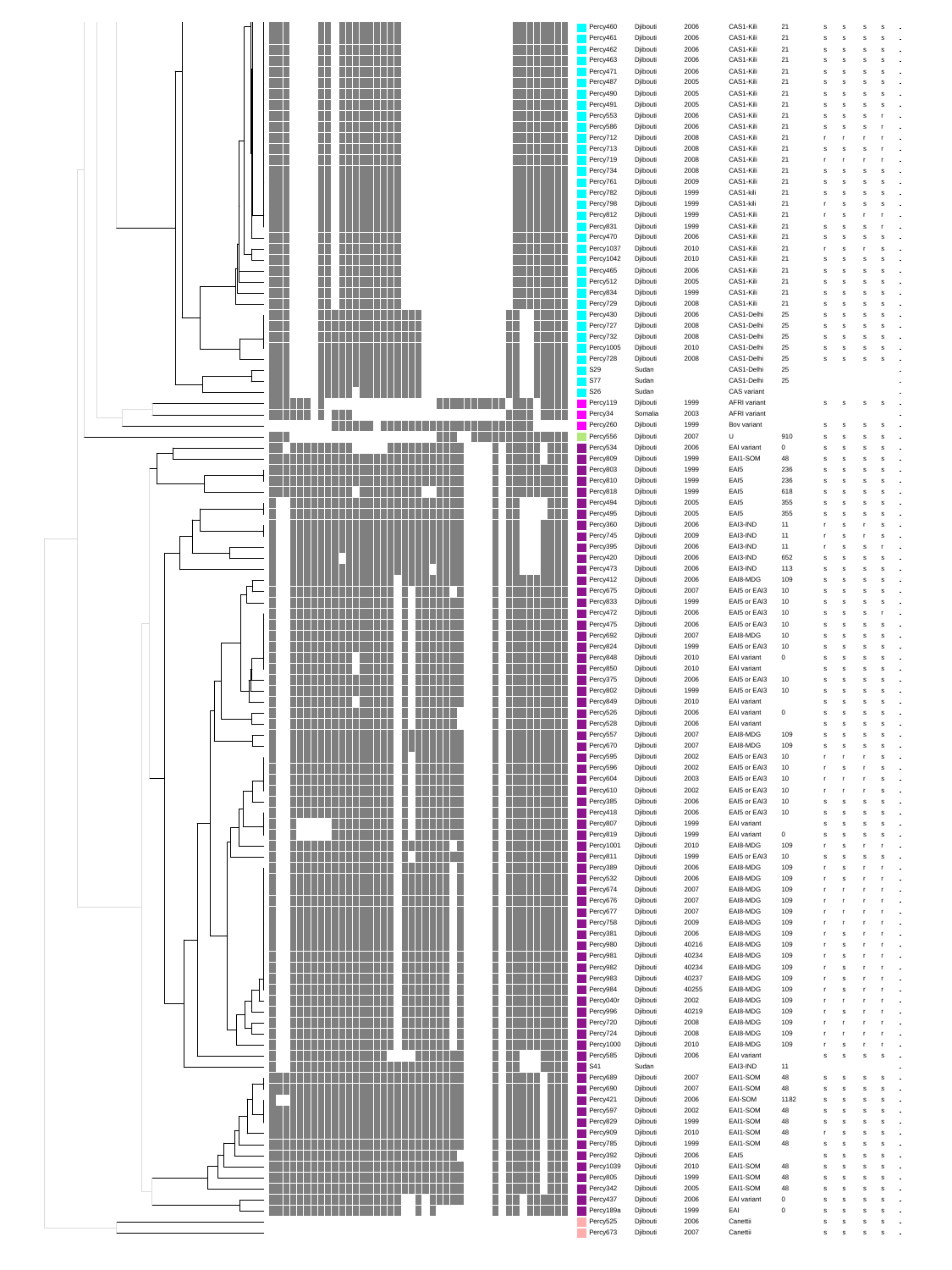

Supplement: Figure S1 — Dendrogram of the 435 isolates based upon MLVA24Orsay. The spoligotype, strain Id, geographic origin, spoligotype clade, year of isolation and antibiotic resistance status are indicated. The color code used is as indicated in Figure 1. The dendrogram is based upon data produced using the previously published MLVA24Orsay assay, the categorical coefficient and UPGMA clustering [13]. Two reference genomes (H37Rv and CDC1551) and two M. canettii isolates, Percy525 and Percy673 are included for comparison. (PPT) [file pone.0052841.s001.ppt]
