# Supplementary material for: Significance of the Identification in the Horn of Africa of an Exceptionally Deep Branching Mycobacterium tuberculosis Clade
Source: PLoS One. 2012 Dec 27;7(12):e52841. doi: 10.1371/journal.pone.0052841 (PMC3531362; doi:10.1371/journal.pone.0052841)
Supplement: Table S2 — MLVA panels associated with internet accessible databases. (DOC) [file pone.0052841.s003.doc]

**Blouin Table S2**

| Locus-position | Popular alias | ETRs^1,2^ | MycoDB.es^5^ | MIRU-VNTR12^1,2,4^ | VNTR_15loci^1^,^3^ | MIRU-VNTR15^1,4^ | MLVA15_China_^1^ | MLVA21_Orsay_^1^ | MLVA24_Orsay_^1^ | MIRU-VNTR24^4^ | MLVA19common^1^* |
| --- | --- | --- | --- | --- | --- | --- | --- | --- | --- | --- | --- |
| ETRA-2165 |  | X | X |  | X | X | X | X | X | X | X |
| ETRB-2461 |  | X | X |  | X |  | X | X | X | X | X |
| ETRC-0577 |  | X |  |  | X | X | X | X | X | X | X |
| ETRD-0580 | MIRU04 | X | X | X | X | X | X | X | X | X | X |
| ETRE-3192 | MIRU31 | X | X | X | X | X | X | X | X | X | X |
| MIRU02-0154 |  |  |  | X | X |  |  | X | X | X | X |
| MIRU10-0960 |  |  |  | X | X | X | X | X | X | X | X |
| MIRU16-1644 |  |  |  | X | X | X | X | X | X | X | X |
| MIRU20-2050 |  |  |  | X | X |  |  |  | X | X | X |
| MIRU23-2531 |  |  |  | X | X |  | X | X | X | X | X |
| MIRU24-2687 |  |  |  | X | X |  |  |  | X | X | X |
| MIRU26-2996 |  |  | X | X | X | X | X |  | X | X | X |
| MIRU27-3006 | Qub5 |  |  | X | X |  | X | X | X | X | X |
| MIRU39-4348 |  |  |  | X | X |  | X | X | X | X | X |
| MIRU40-0802 |  |  |  | X | X | X | X | X | X | X | X |
| Mtub01-0024 |  |  |  |  |  |  |  | X | X |  |  |
| Mtub02-0079 |  |  |  |  |  |  |  | X | X |  |  |
| Mtub04-0424 |  |  |  |  |  | X |  |  |  | X |  |
| Mtub12-1121 |  |  |  |  |  |  |  | X | X |  |  |
| Mtub21-1955 |  |  |  |  |  | X | X | X | X | X | X |
| Mtub29-2347 |  |  |  |  |  |  |  | X | X | X | X |
| Mtub30-2401 |  |  |  |  |  | X | X | X | X | X | X |
| Mtub31-2990 |  |  |  |  |  |  |  |  | X |  |  |
| Mtub34-3171 |  |  |  |  |  |  |  |  |  | X |  |
| Mtub38-3663 |  |  |  |  |  |  |  | X |  |  |  |
| Mtub39-3690 |  |  |  |  |  | X | X | X | X | X | X |
| Qub11a-2163 |  |  | X |  |  |  |  | X | X |  |  |
| Qub11b-2163 |  |  | X |  |  | X |  |  |  | X |  |
| Qub26-4052 |  |  | X |  |  | X |  |  |  | X |  |
| Qub3232-3232 |  |  | X |  |  |  |  |  |  |  |  |
| Qub4156-4156 |  |  |  |  |  | X |  |  |  | X |  |

* the 19 loci shared in assay MLVA24_Orsay_ and MIRU-VNTR24

^1^ panels of loci predefined in the *Mycobacterium tuberculosis* database at [http://mlva.u-psud.fr](http://mlva.u-psud.fr/)

^2^ panels used by SIVIWEB <http://www.pasteur-guadeloupe.fr:8081/SITVIT_ONLINE/>

^3^ selection of 15 loci as used by the TB strain typing database, <http://www.hpa-bionum.org.uk/TBtyping/>

^4^ panels of loci predefined in the MIRU-VNTRplus database [http://www.miru-vntrplus.org](http://www.miru-vntrplus.org/)

^5^ 9 loci used for *Mycobacterium bovis* at <http://mycodb.es/>
