# Supplementary material for: Significance of the Identification in the Horn of Africa of an Exceptionally Deep Branching Mycobacterium tuberculosis Clade
Source: PLoS One. 2012 Dec 27;7(12):e52841. doi: 10.1371/journal.pone.0052841 (PMC3531362; doi:10.1371/journal.pone.0052841)
Supplement: Table S6 — Studies of M. tuberculosis diversity in Eastern Africa countries. (DOC) [file pone.0052841.s007.doc]

Blouin_Table S6

| **Country** | **Strain Nbr** | **Clades** | | | | | | | **Ref** |
| --- | --- | --- | --- | --- | --- | --- | --- | --- | --- |
|  |  | **Modern** | **LAM** | **CAS** | **EAI** | **Beij** | **MANU** | **BOV/AFRI** |  |
|  |  | **T, H, X, S** |  |  |  |  |  |  |  |
| Djibouti | 389 | 53 | 6,25 | 22 | 15 | 2,75 | 0 | 0,7 | This study |
|  |  |  |  |  |  |  |  |  |  |
| Saudi Arabia | 1505 | 27,9 | 7,2 | 22,5 | 13,5 | 4,4 | 2,7 | 0,9 |  |
|  |  |  |  |  |  |  |  |  |  |
| Egypt | 151 | 56.1 | 4.7 | 3.9 |  |  | 26.1 |  |  |
|  |  |  |  |  |  |  |  |  |  |
| Uganda | 344 | 70 | 7 | 7,1 | 0,9 | 1,2 |  |  |  |
|  |  |  |  |  |  |  |  |  |  |
| Tanzania | 130 | 8,4 | 18,4 | 33,8 | 6,2 | 5,4 |  |  |  |
|  |  |  |  |  |  |  |  |  |  |
| Tanzania | 147 | ND | 22 | 37 | 17 |  |  |  |  |
|  |  |  |  |  |  |  |  |  |  |
| Madagascar | 333 | 50,4 | 6 | 9,1 | 23,5 | 4 | 2,7 | 0,3 |  |
|  |  |  |  |  |  |  |  |  |  |
| Zambia | 273 | 19,5 | 41,8 | ND | ND | 7 |  |  |  |
|  |  |  |  |  |  |  |  |  |  |
| Mozambique | 445 | 19,5 | 37 | 2,5 | 29,7 | 7 | 0,7 | 0 |  |
|  |  |  |  |  |  |  |  |  |  |
| South Africa | 252 | 55 | 24 | 2 | 1,2 | 10 | 0,8 |  |  |
|  |  |  |  |  |  |  |  |  |  |
| Sudan | 232 | 11.6 | 2.2 | 55.6 |  | 3 |  |  |  |
|  |  |  |  |  |  |  |  |  |  |
| ND | Not determined | |  |  |  |  |  |  |  |

1. Al-Hajoj SA, Zozio T, Al-Rabiah F, Mohammad V, Al-Nasser M, et al. (2007) First insight into the population structure of *Mycobacterium tuberculosis* in Saudi Arabia. J Clin Microbiol 45: 2467-2473.

2. Helal ZH, Ashour MS, Eissa SA, Abd-Elatef G, Zozio T, et al. (2009) Unexpectedly high proportion of ancestral Manu genotype *Mycobacterium tuberculosis* strains cultured from tuberculosis patients in Egypt. J Clin Microbiol 47: 2794-2801.

3. Asiimwe BB, Ghebremichael S, Kallenius G, Koivula T, Joloba ML (2008) *Mycobacterium tuberculosis* spoligotypes and drug susceptibility pattern of isolates from tuberculosis patients in peri-urban Kampala, Uganda. BMC Infect Dis 8: 101.

4. Kibiki GS, Mulder B, Dolmans WM, de Beer JL, Boeree M, et al. (2007) *M. tuberculosis* genotypic diversity and drug susceptibility pattern in HIV-infected and non-HIV-infected patients in northern Tanzania. BMC Microbiol 7: 51.

5. Eldholm V, Matee M, Mfinanga SG, Heun M, Dahle UR (2006) A first insight into the genetic diversity of *Mycobacterium tuberculosis* in Dar es Salaam, Tanzania, assessed by spoligotyping. BMC Microbiol 6: 76.

6. Ferdinand S, Sola C, Chanteau S, Ramarokoto H, Rasolonavalona T, et al. (2005) A study of spoligotyping-defined *Mycobacterium tuberculosis* clades in relation to the origin of peopling and the demographic history in Madagascar. Infect Genet Evol 5: 340-348.

7. Mulenga C, Shamputa IC, Mwakazanga D, Kapata N, Portaels F, et al. (2010) Diversity of *Mycobacterium tuberculosis* genotypes circulating in Ndola, Zambia. BMC Infect Dis 10: 177.

8. Viegas SO, Machado A, Groenheit R, Ghebremichael S, Pennhag A, et al. (2010) Molecular diversity of *Mycobacterium tuberculosis* isolates from patients with pulmonary tuberculosis in Mozambique. BMC Microbiol 10: 195.

9. Stavrum R, Mphahlele M, Ovreas K, Muthivhi T, Fourie PB, et al. (2009) High diversity of *Mycobacterium tuberculosis* genotypes in South Africa and preponderance of mixed infections among ST53 isolates. J Clin Microbiol 47: 1848-1856.

10. Sharaf Eldin GS, Fadl-Elmula I, Ali MS, Ali AB, Salih AL, et al. (2011) Tuberculosis in Sudan: a study of *Mycobacterium tuberculosis* strain genotype and susceptibility to anti-tuberculosis drugs. BMC Infect Dis 11: 219.
